# Addressing Dynamics at Catalytic Heterogeneous Interfaces with DFT-MD: Anomalous Temperature Distributions from Commonly Used Thermostats

**DOI:** 10.1021/acs.jpclett.2c00230

**Published:** 2022-03-17

**Authors:** Ville Korpelin, Toni Kiljunen, Marko M. Melander, Miguel A. Caro, Henrik H. Kristoffersen, Nisha Mammen, Vesa Apaja, Karoliina Honkala

**Affiliations:** † Department of Chemistry, Nanoscience Center, University of Jyväskylä, P.O. Box 35 (YN), FI-40014 Jyväskylä, Finland; ‡ Department of Electrical Engineering and Automation, Aalto University, FIN-02150 Espoo, Finland; ¶ Department of Chemistry, University of Copenhagen, 2100 Copenhagen Ø, Denmark; § Department of Physics,Nanoscience Center, University of Jyväskylä, P.O. Box 35 (YN), FI-40014 Jyväskylä, Finland

## Abstract

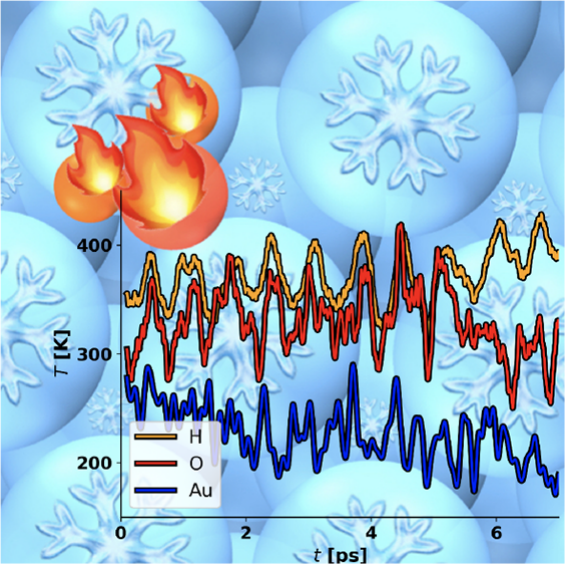

Density functional theory-based molecular dynamics (DFT-MD) has been widely used for studying the chemistry of heterogeneous interfacial systems under operational conditions. We report frequently overlooked errors in thermostated or constant-temperature DFT-MD simulations applied to study (electro)catalytic chemistry. Our results demonstrate that commonly used thermostats such as Nosé–Hoover, Berendsen, and simple velocity-rescaling methods fail to provide a reliable temperature description for systems considered. Instead, nonconstant temperatures and large temperature gradients within the different parts of the system are observed. The errors are not a “feature” of any particular code but are present in several *ab initio* molecular dynamics implementations. This uneven temperature distribution, due to inadequate thermostatting, is well-known in the classical MD community, where it is ascribed to the failure in kinetic energy equipartition among different degrees of freedom in heterogeneous systems (Harvey *et al*. J. Comput. Chem.1998, 726−740) and termed the flying ice cube effect. We provide tantamount evidence that interfacial systems are susceptible to substantial flying ice cube effects and demonstrate that the traditional Nosé–Hoover and Berendsen thermostats should be applied with care when simulating, for example, catalytic properties or structures of solvated interfaces and supported clusters. We conclude that the flying ice cube effect in these systems can be conveniently avoided using Langevin dynamics.

Molecular dynamics (MD) simulations in the canonical, fixed *NVT* ensemble are a powerful way to study thermodynamic properties of condensed phases. In past years, density functional theory (DFT) based MD simulations have been widely applied to study interfacial and heterogeneous systems such as metal–water interfaces and supported nanoclusters relevant for heterogeneous (electro)catalytic processes.^1,2^ These studies require substantial computer resources for well-converged simulation results and are often considered to provide an unbiased benchmark-quality description of such complex interfaces with warranted accuracy.^3^

From a technical perspective, sampling the *NVT* ensemble requires the use of thermostats to achieve constant temperatures in simulations. A thermostat introduces an approximate, preferably nonintrusive, coupling of the system to a fictitious heat bath, and several types of thermostats have been developed^4^ for this purpose over the years. However, the vast majority of existing DFT-MD studies of heterogeneous and interfacial systems have employed Nosé–Hoover thermostats,^3,5−27^ the Berendsen^28−36^ or even simple velocity-rescaling thermostats,^37^ or Langevin dynamics.^38−42^ All the referenced works have focused on either solvated interfaces in (electro)catalytic systems or on (supported) nanocluster catalysts which exemplify the wide adoption of simple, single-chain Nosé–Hoover,^43,44^ Berendsen,^45^ or simple velocity-rescaling thermostats by the catalysis community. Another common aspect is that these studies represent high-profile research, which has established the use of DFT-MD in providing crucial atomistic insight into (electro)catalytic processes.

While these methods used in the above-mentioned studies can faithfully reproduce the *average temperature* of the system correctly, we have observed that both the Nosé–Hoover and Berendsen thermostats fail to provide a *uniform temperature* throughout the simulation cell. Instead, substantial *temperature gradients* exist in the simulated systems, for example, between water and a metallic surface, or an active metal catalyst and an oxide support, and even within bulk water, where rotations, vibrations, and translations exhibit different thermal energies.^46^ Similar anomalies are likely present in several DFT-MD simulations of interfacial systems. Therefore, some previous results obtained with DFT-MD and the Berendsen or Nosé–Hoover thermostats should be approached cautiously and re-examined with other thermostats or Langevin dynamics.

Previously such anomalies have been demonstrated and thoroughly analyzed for classical MD simulations with weak velocity-rescaling thermostats such as the Berendsen thermostat. This led to the identification of the “flying ice cube” effect where the kinetic energy is incorrectly partitioned within the system and becomes transferred from high-frequency modes to low-frequency modes.^47−50^ Another manifestation of similar issues is the “hot-solvent/cold-solute” problem,^51^ which was resolved by using separate Nosé–Hoover chains for the solute and the solvent.^52^ The flying ice cube effect can be reduced, for example, by increasing the system size, decreasing the time step, or adjusting the thermostat,^47^ but these measures would further increase the already high cost of DFT-MD simulations. The presence of temperature gradients and incorrect temperature partitioning has most often been observed and discussed for the Berendsen thermostat, but in principle it is possible to have similar issues also with other thermostats, such as Nosé–Hoover.^51,53−55^ The issue is not only theoretical but also affects the observed thermodynamics, sampling and ergodicity, temporal dynamics, and computed expectation values.^47,52,56^ As highlighted in a recent study,^57^ accurate modeling of both the average temperature and its fluctuations is important to reconcile computed and measured adsorption energies.

The present Letter aims to illustrate, to our knowledge for the first time, that the widely used single-chain Nosé–Hoover and Berendsen thermostats can show extreme features of the flying ice cube effect in heterogeneous (electro)catalysis DFT-MD simulations, even when tight convergence criteria are used for self-consistency. We show that temperatures or kinetic energy partitioning from these thermostats do not follow their expected average values even with improved convergence and that the deviations escalate with typical convergence criteria. We demonstrate that extremely well-converged calculations are needed to obtain the correct kinetic energy partitioning even in a simple diatomic condensed phase system (N_2_); otherwise, the energy is incorrectly partitioned between vibrations, rotations, and translations. For more complex systems, we show that temperature gradients persist even when the energies are converged to 10^–5^–10^–7^ eV accuracy for an electrochemical interface (a water–Au(111) interface) and for a heterogeneous catalyst model (a zirconia-supported Pt cluster). Furthermore, we demonstrate that using typical energy convergence criteria of 10^–3^–10^–4^ eV can lead to incorrect temperature/kinetic energy distributions between different kinds of atoms even in the same phase.

Several different DFT softwares, including GPAW^58,59^ with ASE,^60^ VASP,^61^ CP2K,^62^ and Quantum ESPRESSO (QE),^63^ were used to carry out DFT-MD simulations for the considered interfacial model systems shown in Figures S1 and S2. Depending on the availability of different thermostats in these codes, we examined the performance of single-chain Nosé–Hoover^64−66^ and Berendsen^45^ thermostats and the Langevin dynamics.^4^ Various energy and density convergence criteria were tested to address the coupling between accurate energies (forces) and the performance of the temperature controls as detailed in the Supporting Information.

The time step of 1 fs was considered a valid starting point for typical DFT-MD simulations. The thermostat parameters were chosen as representative values to illustrate the flying ice cube effects. The influence of temperature gradients on dynamical and thermodynamics values was probed by computing entropies for the simulated trajectories using velocity–velocity correlation functions within the 2PT formalism^67^ as implemented in the DoSPT code.^46^ The DoSPT program distinguishes between translational, rotational, and vibrational degrees of freedom (DoF) in the kinetic energy partitioning. For extracting dynamic quantities, such as correlation functions, from Langevin dynamics, the selection of a friction parameter is important to achieve efficient thermalization without disturbing the system dynamics too much.^56^ Therefore, a rather small friction coefficient was applied and we expect that the accuracy of the computed velocity autocorrelation functions is not substantially affected by this choice. A more comprehensive overview on the computational methods is provided in the Supporting Information.

We start with presenting the results for 64 N_2_ molecules in a fully periodic cubic 27 nm^3^ simulation cell corresponding to supercritical fluid conditions. This homogeneous system allows the evaluation of thermostat performance in a simple case where the kinetic energy partitioning is easy to define. The choice of the test system was motivated by refs (47 and 48), in which the kinetic energy partitioning in ethane was considered in a similar manner.

The DFT-MD simulations were started with the 18.5 ps Langevin dynamics run to equilibrate the N_2_ system at 300 K. Density of states analysis shows (Table S1) that the dynamics maintains the correct equipartition between center-of-mass (C.M.) translations (192 degrees of freedom), rotations (128), and vibrations (64) within acceptable limits. This demonstrates that Langevin dynamics performs well even with the loose GPAW-default density convergence criterion of 10^–4^ electrons per valence electron (e/v.e.), which amounts to 0.064 electrons in the present system. After equilibration, we switched on the Nosé–Hoover thermostat with a coupling time constant of 50 fs and followed the time evolution of the kinetic energy partitioning for 10 ps. Panels in Figure 1 show this evolution for three distinct values of the density convergence criterion given in the figure caption that converge the total energies at least to 10^–4^, 10^–6^, and 10^–8^ eV.

**Figure 1:**
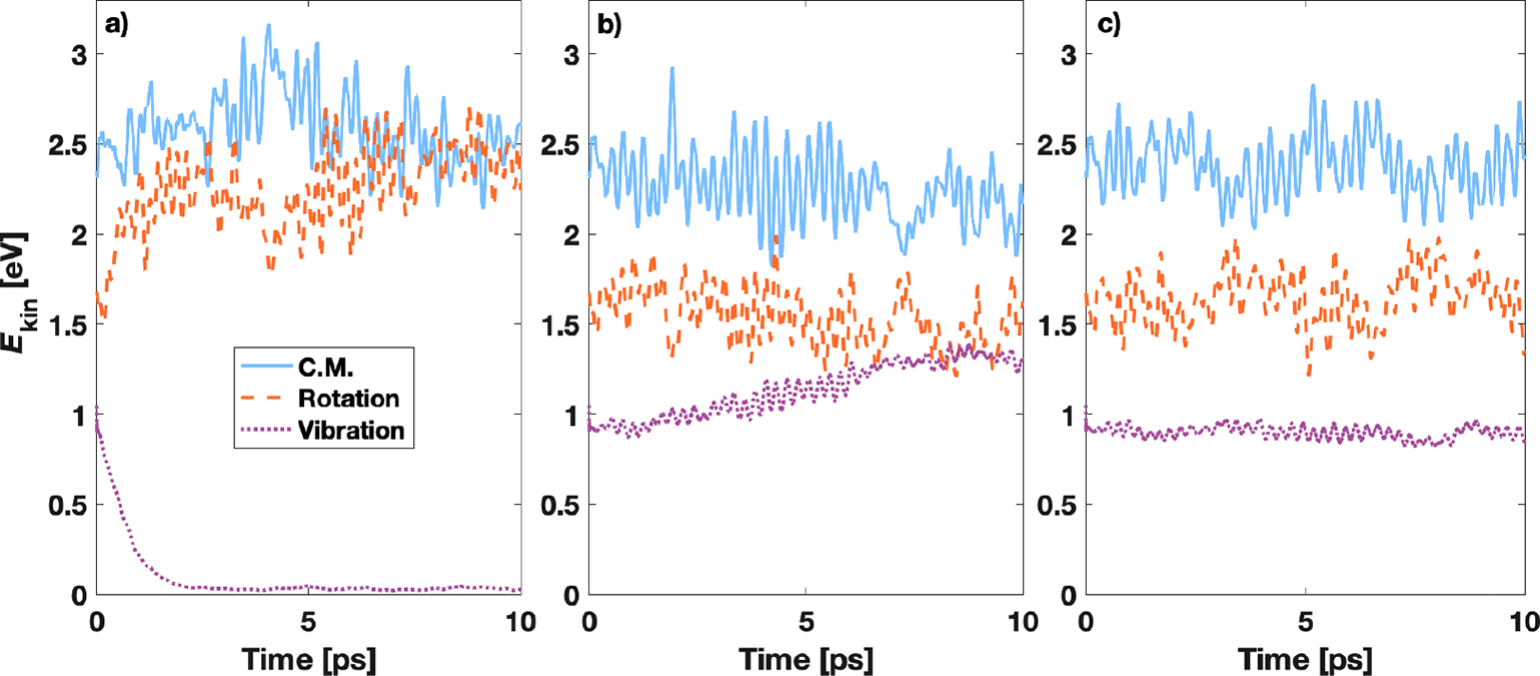
Kinetic energy partitioning for the 64 N_2_ molecules thermostated by Nosé–Hoover at 300 K with density convergence criteria of (a) 10^–4^ e/v.e., (b) 10^–5^ e/v.e., and (c) 10^–6^ e/v.e. The DFT-MD trajectories were obtained using ASE/GPAW code. Moving averages of trailing 0.1 ps are plotted for C.M. translations, rotations, and vibrations.

From Figure 1 it is clear that insufficient convergence together with the Nosé–Hoover thermostat leads to unwanted kinetic energy partitioning in the N_2_ system. Using the loosest criterion of 10^–4^ e/v.e., the vibrational motions almost completely freeze after 2 ps, while the rotational and translational energies become roughly equal with each other. The same anomaly occurs by using the Berendsen thermostat (shown in Figure S3). The distribution can be somewhat improved by tightening the density convergence by an order of magnitude to 10^–5^ e/v.e, but now the vibrational component gains too much energy. Note that the average temperature is always correct and around the wanted 300 K, but the kinetic energy is incorrectly distributed. The proper equipartition for the diatomic system is maintained only by tightening the density convergence by yet another order of magnitude, down to 10^–6^ e/v.e. (shown in Figure 1 c). This density convergence reduces the energy change between electronic iterations below 10^–8^ eV, which is an unusually tight energy convergence criterion rarely achieved in large-scale DFT-MD simulations. The resulting energy partitioning compares to that of the Langevin dynamics reasonably well, as shown in Figure S4. In addition, we employed the CP2K software to verify that with tight, 10^–6^ Ha convergence, the quality of energy partitioning is similar between the Langevin and the Nosé–Hoover simulations (please see Figure S5). We also compared the Berendsen thermostat to the simple velocity rescaling using the QE code (Figure S6). In these simulations, the loose convergence manifests itself in energy anomalies despite the strict control over the total kinetic energy and temperature. Here, the energy distribution is very different from the above ASE/GPAW Nosé–Hoover and Berendsen cases.

All these examples demonstrate that the force inaccuracy, originating from insufficient energy convergence, intensifies the tendency to exhibit the flying ice cube effect even when the convergence criteria are tighter than the default values. We note that in systems with frequent collisions, the dynamical energy redistribution may level out the energy partitioning and counteract the flying ice cube effect. However, the present N_2_ test system is relatively sparse and thus serves as an example to highlight the role of the molecule–thermostat coupling.

Metal–water interfaces are ubiquitous and of great significance in the field of (electro)catalysis. Atomic level knowledge of the dynamical properties of these interfaces is essential for fundamental understanding of (electro)catalytic reaction mechanisms and design of advanced (electro)catalysts for efficient conversion of energy and molecules. Before considering the performance of different thermostats and software on probing the dynamic evolution of the metal–water interface, bulk water was studied. It is homogeneous but denser and more complex than the model N_2_ system. The bulk water model consists of 64 molecules in a (1.242 nm)^3^ cube. The simulations were carried out with the ASE/GPAW software and the Nosé–Hoover thermostat, Langevin dynamics, or *NVE* dynamics using 10^–4^ e/v.e. density convergence and 2 u for hydrogen mass to ensure the same 1 fs time step as for N_2_. The instantaneous temperature given in Figure S7 and the density of state plot in Figure S8 show that Langevin dynamics clearly outperforms the Nosé–Hoover thermostat. Furthermore, the *NVE* dynamics runs revealed the necessity to tighten the convergence to 10^–6^ e/v.e. (below 10^–8^ eV) to obtain energy conservation and proper partitioning (see Figure S9). The DoS plot (Figure S8) illustrates the flying ice cube effect by showing a thermostat-dependent misproportion of high-frequency vibrational modes and low-frequency translations and rotations.

The presence of metal surface modifies the water degrees of freedom and the entropy reflecting the mobility constraint due to the surface, and collisions with surface atoms introduce an energy-exchange mechanism, both dependent on the accuracy of the kinetic energy partitioning. We performed DFT-MD simulations for a solvated Au(111) surface having 32 water molecules and 24 mobile Au atoms and employing different software. The most extensive, over 100 ps, simulations were carried out with the VASP code using an energy convergence criterion of 10^–4^ eV. Figure 2 illustrates the kinetic energy partitioning and allows a comparison between the Langevin and Nosé–Hoover runs. The plotted curves again represent center of mass, rotational, and vibrational motions, as well as the total sum of the energy components. For Langevin dynamics, rotations and vibrations fully overlap at the energy of ∼1.4 eV as expected, and translations are offset by approximately 1.0 eV because of the mobile surface Au atoms. The total kinetic energy is 5.12 eV, and all its components faithfully average to the target temperature of 330 K. With the Nosé–Hoover thermostat, the oscillations of total energy efficiently level out with the 0.1 ps smoothing. However, the inability of the thermostat to work properly is demonstrated by the kinetic energy components which average to 360 K (2.61 eV) for translations, 329 K (1.36 eV) for rotations, and 284 K (1.17 eV) for vibrations. In contrast to Langevin dynamics, the thermostated energies also exhibit drifting from the average values.

**Figure 2:**
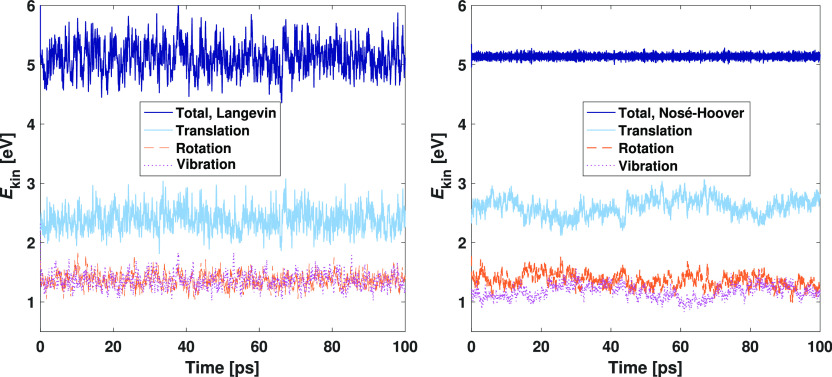
Kinetic energy partitioning into translational, rotational, and vibrational degrees of freedom in VASP simulations of the Au(111)–H_2_O system with Langevin dynamics (left) or Nosé–Hoover thermostat (right) targeted at 330 K. For clarity, the moving averages of trailing 0.1 ps periods are plotted.

As the VASP DFT-MD simulations for the Au(111)–H_2_O were long enough, they allow reliable evaluation of thermodynamic properties like entropy (*S*) and the impact of incorrect temperature partitioning on a thermodynamic observable. The number of degrees of freedom provides a convenient measure for the accuracy of kinetic energy partitioning. The 24 mobile gold atoms should exhibit 72 (3 × 24) DoF in the translational mode, while the total number of 288 DoF of the 32 water molecules should be evenly distributed in 96 translations, 96 rotations, and 96 vibrations. These numbers display as areas under the density of states (DoS) curves obtained from the DoSPT program. Figure 3 shows the separate DoS plots for the water molecules and surface gold atoms. The most notable difference between the Langevin and Nosé–Hoover results is seen in the Au translations, for which Nosé–Hoover gives a peaked structure while Langevin shows a smooth curve. A similar excess in density is also present at the translational frequencies of water. The difference in other water modes appears almost indistinguishable. On closer inspection, comparing Langevin DoS curves to Nosé–Hoover DoS curves shows slight decrease in density of the high-frequency vibrational mode and accordingly slight increase in the low-frequency vibrational mode in the Nosé–Hoover DoS curve. The observed energy transfer is in line with the description of the flying ice cube effect.^48^

**Figure 3:**
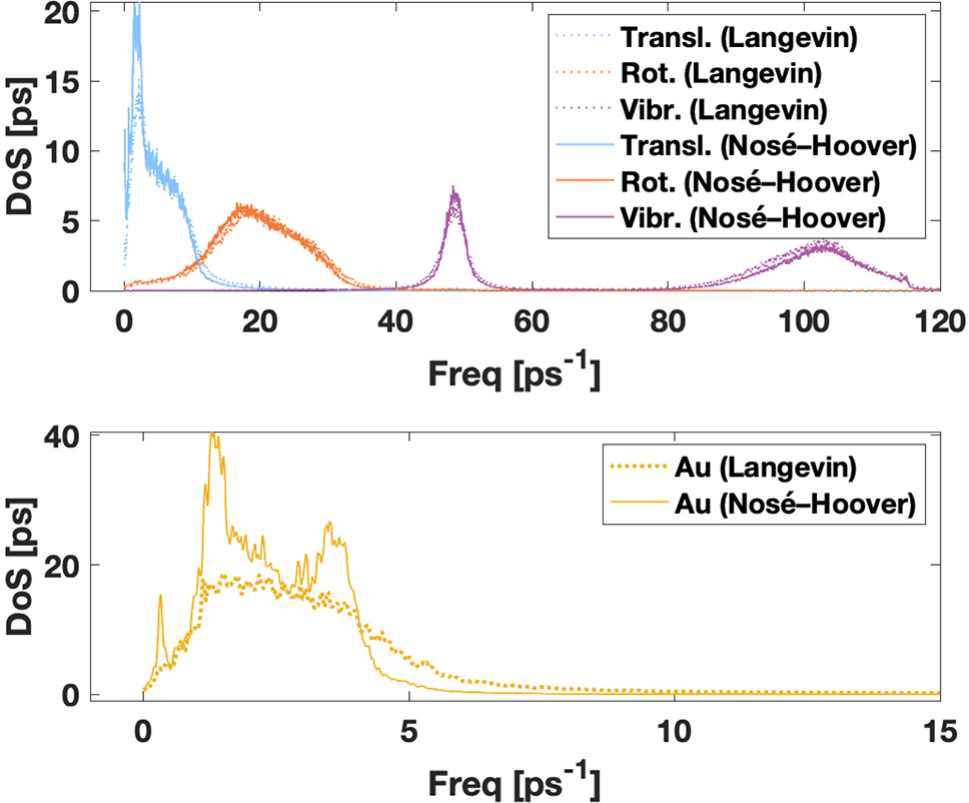
Density of states plot for the H_2_O–Au(111) system presenting translational, rotational, and vibrational degrees of freedom of water molecules (upper) and of Au atoms (lower).

The DoF numbers deviate more clearly. While Langevin produces them correctly, giving 72 translation DoF to the Au slab and 96 DoF to each water mode, Nosé–Hoover produces 82 Au translations, and the water modes are 101, 96, and 83 DoF for translations, rotations, and vibrations, respectively. The entropy value computed from the Langevin dynamics is *S* = 50 J K^–1^ mol^–1^ for H_2_O, whereas the Nosé–Hoover gives 61 J K^–1^ mol^–1^. The 20% entropy difference principally reflects the varying number of translational DoF and thus entropies for the translational Au part expectedly differ, being 48 J K^–1^ mol^–1^ and 63 J K^–1^ mol^–1^, respectively.

Next, we compared the performance of different DFT codes and analyzed the instantaneous temperatures for solvated Au(111) from the Langevin dynamics and the Nosé–Hoover thermostat simulations. The DFT packages VASP, GPAW, and CP2K were used. Table 1 summarizes the instantaneous temperatures and shows that Langevin dynamics produces the correct average temperature (330 K for VASP and CP2K, 300 K for ASE/GPAW) in the entire system and also all the atoms are reasonably close to the average temperature. Whereas the Nosé–Hoover thermostat satisfactorily keeps the expected average temperature (350 K ASE/GPAW, 330 K others), the different atoms show large temperature variations. We observed up to 100 K differences between water and surface temperatures, although the DFT-MD calculations were tightly converged (see Figures S10−S12). Interestingly, depending on the code, the water molecules may be either hotter (CP2K) or colder (VASP) than the Au surface despite the very similar computational setups. The CP2K Nosé–Hoover data has, for example, high-temperature hydrogen atoms, which are accompanied by a marked increase in the vibrational stretching mode intensity (see Figures S14 and S15), whereas other DoF are reduced with respect to the CP2K Langevin data, pointing toward the opposite^68^ or “inverted flying ice cube effect”. In the VASP Nosé–Hoover simulations, the Au atoms, in turn, appear overheated, which indicates energy transfer from high-frequency vibrational modes to translational modes due to the conventional flying ice cube effect. Figure 2 reveals that the inverted flying ice cube may change to the conventional one and vice versa on a time scale of tens of picoseconds. Altogether, these results highlight the fact that the kinetic energy partitioning for Nosé–Hoover trajectories depends sensitively on the DFT-MD software, making it challenging to identify the underlying reason for the incorrect kinetic energy distribution between the two subsystems of a metal–water interface.

**Table 1:**
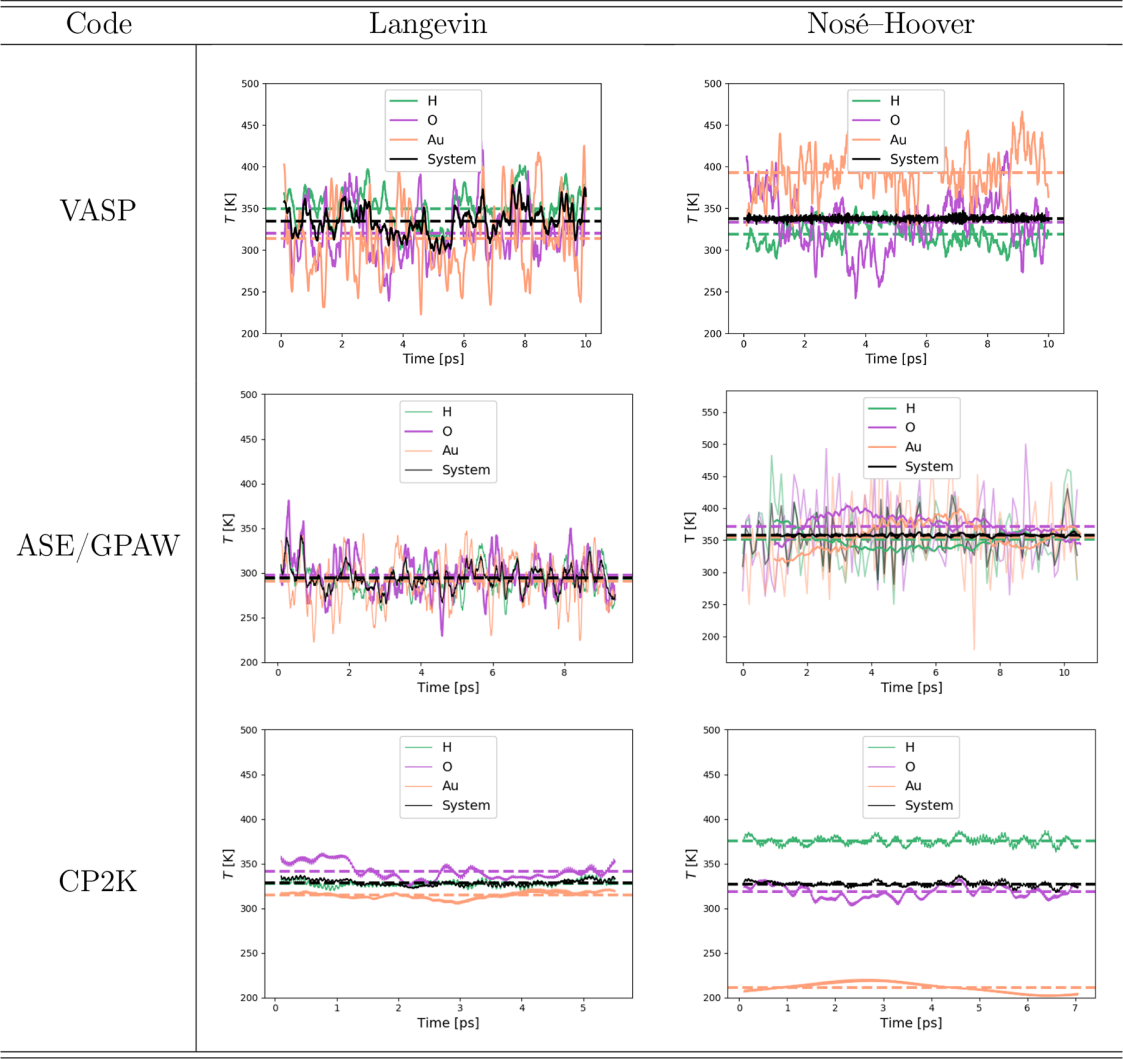
Comparison of Atom-Specific Instantaneous Temperatures for the Solvated Au(111) Surface Obtained Using Langevin Dynamics and Nosé–Hoover Thermostat with Different DFT-MD Codes^a^

a The inserted figures show 0.1 ps moving averages along with the full averages from the presented time slice.

The spurious temperature distribution from Nosé–Hoover dynamics was aggravated when the convergence criteria were left looser, as shown in Figure S10 for ASE/GPAW trajectories. Despite strayed atomic constituents, the average temperature is correct, which hides the erroneous performance. The thermostat time scale may also have an effect on the temperature distribution, and in Figure S11 we show that with a tighter coupling, that is, using a smaller time scale parameter, the system achieved thermalization faster and more correctly. It should, however, be noted that too aggressive thermostatting can disturb the system dynamics and, for example, impact the calculation of correlation functions.

In addition to our own data, we also analyzed temperature distributions from two examples of recently published electrochemical interface studies, where computational data were freely available. In the first example, solvated Cu surfaces with and without adsorbed hydrogen atoms were simulated using the Berendsen thermostat.^36^ The temperature analysis is presented in Figure S17, and it reveals that the temperature distributions of the hydrogen and oxygen atoms in the water solvent depend strongly on the surface structure. However, it is the adsorbed H atoms which suffer from a more striking temperature variation and appear up to 500 K hotter than other species. If a H diffusion constant were computed from these results, the atom would appear anomalously mobile. In the second example, a solvated Au surface was studied in the presence of Li^+^ ions with and without an adsorbed CO_2_, using the Nosé–Hoover thermostat.^26^ The temperature analysis given in Figure S18 demonstrates significant temperature gradients and differences. In particular, the temperature of both the Li^+^ ions and the CO_2_ molecule are, for the major part of the 2 ps production run, far from the target temperature of 300 K and thermodynamic equilibrium.

Finally, we discuss a metal–oxide interfacial system. Oxide-supported metals are typical examples of heterogeneous catalysts, and the metal–oxide interface can provide a rich variety of possible reaction sites. An accurate picture of the interfacial structure and dynamics is necessary to understand the nature of these sites. Here, we use Pt_13_ on *m*-ZrO_2_(1̅11) as our model system. The globally optimized geometry determined previously in the group was taken as an initial structure for the DFT-MD simulation.^69^ The isomerization and diffusion of supported clusters are highly relevant phenomena in determining the structure and activity of a heterogeneous catalyst, and DFT-MD can be used to study these in atomic detail.^2^ To attain reliable results that properly describe the system under *NVT* conditions, the correct kinetic energy distribution between the cluster and the oxide is crucial. If the effective temperature of the cluster is too low/high, this leads to too low/high isomerization and diffusion rates and the biased distribution of the cluster isomer structures skewing the observed cluster ensemble. The importance of knowing properties of the entire cluster ensemble, rather than just the most stable structure, has been increasingly recognized recently,^2^ and a biased view of the ensemble could lead to misrepresented catalytic properties.

We started an ASE/GPAW Nosé–Hoover run from a Langevin-equilibrated structure, using the default GPAW eigenstate and density convergence criteria with a tightened energy criterion of 10^–7^ eV/v.e. (in absolute terms, ca. 2 × 10^–4^ eV for this system). The left panel in Figure 4 shows that the smoothed average temperature of the Pt cluster increases almost linearly at the rate of ∼60 K/ps, reaching over 700 K after 7 ps had passed. Correspondingly, the zirconia surface cools to maintain the average temperature at 300 K. The temperature distribution is uneven also within the ZrO_2_ surface, where the Zr cations heat to ∼350 K while the O anions cool to ∼200 K. This unphysical temperature behavior clearly demonstrates that tighter-than-usual SCF convergence should be enforced when running DFT-MD.

**Figure 4:**
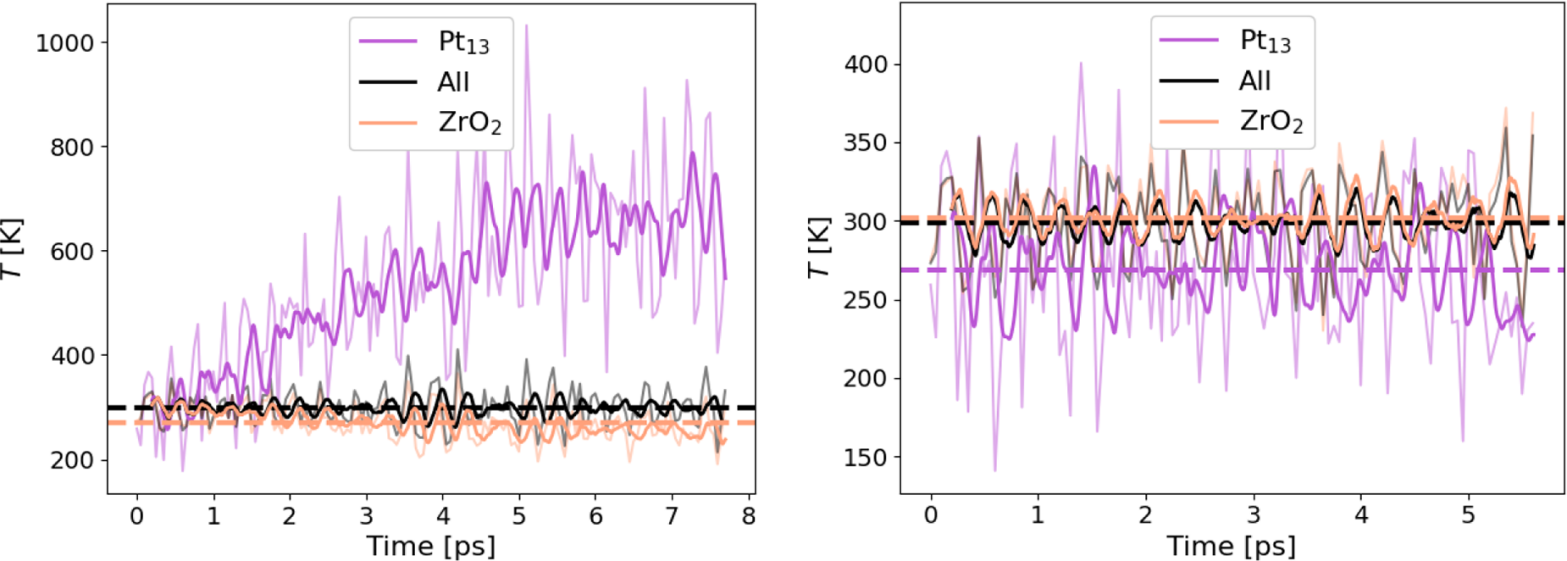
Surface and cluster temperatures for the Pt_13_/ZrO_2_ system obtained with Nosé–Hoover thermostat in GPAW. Left: energy convergence set at 10^–7^ eV/v.e. Right: density convergence set at 10^–6^ e/v.e. Thick curves correspond to 200 fs moving averages.

Therefore, we further tightened the convergence by setting the density criterion to 10^–6^ e/v.e. (ca. 10^–8^ eV in practice), which produced a correct kinetic energy partitioning in the N_2_ system and improves the description of Pt_13_/ZrO_2_ system as well, as seen in the right panel of Figure 4. In comparison to the previous, loosely converged run, the Pt cluster now cools compared to the ZrO_2_ surface, which instead heats up. The cooling effect is much smaller in magnitude, however, and the smoothed average of the cluster temperature hovered around 270 K during the 5 ps run. Still, the results show that even an unusually tight convergence criterion does not guarantee a uniform temperature throughout the system; we presume the flying ice cube effect is likely behind the observed violation of equipartition.

In conclusion, we have shown that combining DFT-MD with widely applied thermostats cannot provide a uniformly constant temperature description of interfacial systems relevant to heterogeneous catalysis and electrocatalysis. Using the DoSPT analysis, we observed that even very tightly converged DFT calculations combined with Nosé–Hoover or Berendsen thermostats lead to temperature gradients between metal and water or between a support and a catalyst. The incorrect temperature description of common thermostats is due to the incorrect kinetic energy distribution between different subsystems present in the simulation—a physical anomaly known as the “flying ice cube” effect. It is present even when we use extremely tight convergence criteria and is further exacerbated if the atomic forces are inaccurate as a result of poor energy convergence.

Our study conclusively demonstrates that current gold standard methods combining DFT-MD and Nosé–Hoover/Berendsen thermostats yield an erroneous thermodynamic description of heterogeneous systems and that previous benchmark simulations are likely subject to significant inaccuracies due to the presence of unphysical temperature gradients. Langevin dynamics instead provides a uniform constant temperature throughout the system, but the friction coefficient needs to be carefully chosen to balance between correct kinetic energy partitioning and minimal disturbance to the system dynamics. Also advanced methods such as the Bussi–Donadio–Parrinello^70^ or Nosé–Hoover chain^71^ thermostats are known^47^ to alleviate the flying ice cube effect, but they are not widely available in common DFT-MD codes such as VASP and QuantumEspresso or through the ASE interface. Overall, the results and analysis presented herein serve as a reminder that accurate and thermodynamically consistent DFT-MD simulations of heterogeneous interfaces require careful testing and validation of both the DFT and MD parts of the method.

## Supporting Information Available

More details on different simulation methods, additional computational results to complement the analysis data provided in the Letter, and the analysis of recent literature data on solvated copper and gold interfaces (PDF)
Transparent Peer Review report available (PDF)

## Notes

Most analyzed and presented data and used scripts are available through the Etsin fairdata service at 10.23729/00d5f9e0-a188-4512-ac95-2069a5aaf6ef. The VASP data was not uploaded because of its extremely large size but can be obtained from the authors.
