# Supplementary material for: Addressing Dynamics at Catalytic Heterogeneous Interfaces with DFT-MD: Anomalous Temperature Distributions from Commonly Used Thermostats
Source: J Phys Chem Lett. 2022 Mar 17;13(11):2644–52. doi: 10.1021/acs.jpclett.2c00230 (PMC8959310; doi:10.1021/acs.jpclett.2c00230)
Supplement: Supplementary file 1 — jz2c00230_si_001.pdf [file jz2c00230_si_001.pdf]

Supplementary Information for

Addressing dynamics at catalytic heterogeneous interfaces with DFT-MD: anomalous temperature distributions from commonly used thermostats

Ville Korpelin,[†,‖] Toni Kiljunen,[†,‖] Marko M. Melander,[†,‖] Miguel A. Caro,[‡] Henrik H. Kristoffersen,[¶] Nisha Mammen,[§] Vesa Apaja,[§] and Karoliina Honkala[*,†]

†Department of Chemistry,Nanoscience Center, University of Jyväskylä, P.O. Box 35 (YN), FI-40014 Jyväskylä, Finland

‡Department of Electrical Engineering and Automation, Aalto University, FIN-02150 Espoo, Finland

¶Department of Chemistry, University of Copenhagen, 2100 Copenhagen Ø, Denmark

§Department of Physics,Nanoscience Center, University of Jyväskylä, P.O. Box 35 (YN), FI-40014 Jyväskylä, Finland

‖These authors contributed equally

E-mail: karoliina.honkala@jyu.fi

More details on different simulation methods are given here for each of the studied model systems N_2, bulk water, Au(111)-H_2O, and Pt_{13}/ZrO_2. This is followed by the additional computational results to complement the analysis data provided in the main manuscript. We also include the analysis of recent literature data on solvated copper[1] and gold[2] interfaces.

Simulated model systems. The N_2 simulation box is shown in Fig. S1. The system consists of 64 N_2 molecules in a cubic box of the length 30 Å, which represents supercritical fluid conditions at 110 g/dm^3 (the ideal gas pressure amounts to 9.8 MPa). For comparison, the simulation box for bulk water is shown on the right. Here, the box length is 12.42 Å corresponding to a 1 kg/dm^3 density. These model systems were used to compare the properties and behavior of two molecular bulk systems. In addition, investigation of the water properties resulting from the DFT-MD thermostats is useful for interpreting the metal-water interface system addressed below.

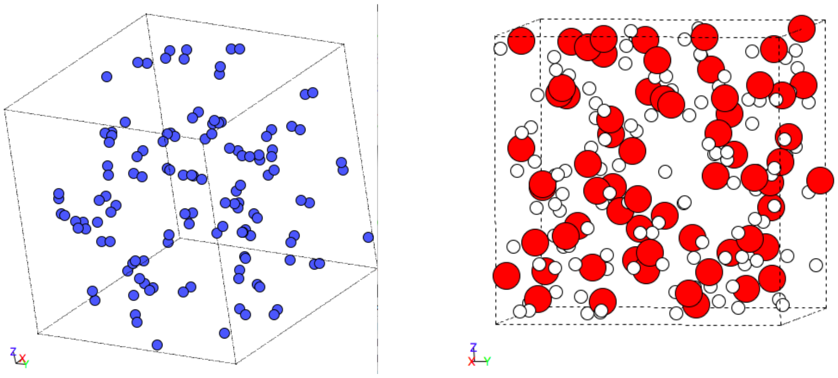

Figure S1: Simulation boxes for the 64-molecule N_2 (left) and H_2O (right) DFT-MD evaluations.

The interfacial Au(111)-H_2O system is shown in the left panel of Fig. S2. The four layers of metal are cut to expose the (111) surface at the interface, and the 4-layer water film ($\sim$ 10 Å) is considered thick enough[3] for modeling the first few solvation layers. The second investigated heterogeneous system is a supported nanocluster, Pt_{13}/ZrO_2, shown on the right in Fig. S2. The initial Pt_{13}/ZrO_2 structure is taken from a previous study, where the Pt_{13} cluster was globally optimized on a m-$ZrO_2(11\bar{1})$ surface.[4]

GPAW Setups. The GPAW DFT code (GPAW version 20.1.0, ASE version 3.19.0)[5–7] was used for all the present model systems from N_2 molecules to the zirconia-supported Pt_{13} cluster. The default convergence tolerances for self consistency in GPAW are $5 \cdot 10^{-4}$ eV per valence electron (eV/v.e.) for maximum total energy change and 10^{-4} electrons per valence electron (e/v.e.) for maximum integral of absolute density change. The absolute value for

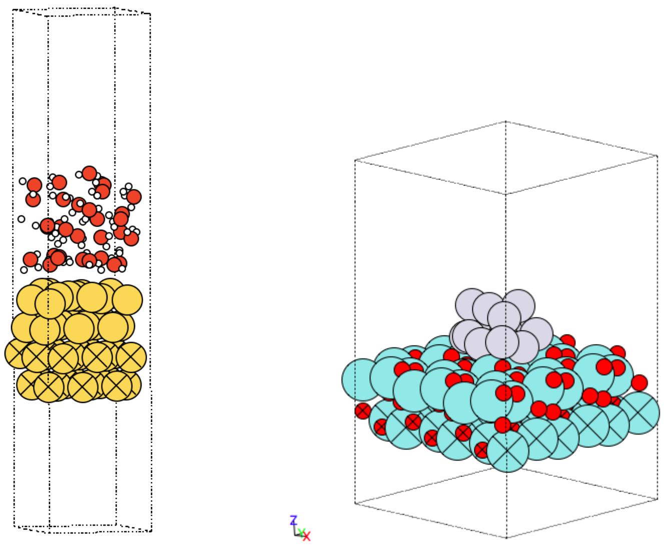

Figure S2: Simulation boxes for the Au(111)-H_2O interfacial and the supported metal cluster systems. The left side model includes 48 Au atoms and 32 water molecules. The right side includes a supporting two-layer surface slab of zirconia and a cluster of 13 Pt atoms.

convergence thus depends on the number of electrons in the system.

N_2 model system. The bulk N_2 was simulated with 3D periodic conditions using the LCAO mode (DZP basis) and the PBE functional.[8] The density was represented on a grid with a spacing of 0.2 Å. The convergence criteria were set for self consistency of the electronic density, at the range from 10^{-3} to 10^{-7} e/v.e. The value of electronic density convergence overrules the default value for energy convergence criterion. For example, the 10^{-5} e/e.v. density convergence criterion for the model system translates to better than 10^{-6} eV total energy convergence in practice.

Maxwell–Boltzmann distribution of velocities was used to initiate the thermalization which was continued for 18.5 ps with Langevin dynamics. The production runs were continued for 10–20 ps with Berendsen and Nosé–Hoover thermostats known to be susceptible to the flying ice cube effect.[9] Both methods are implemented in ASE in different modules used for integrating the equations of motion. The `ase.md.npt.NPT` thermostat was used for Nosé-Hoover (barostat disabled), `ase.md.nvtberendsen.NVTBerendsen` for Berendsen, and `ase.md.langevin.Langevin` for Langevin runs. All the MD propagators used a time

step of 1 fs, with a target temperature of 300 K. The default coupling parameters for the thermostats were set as follows: `ttime = 50 fs` for Nosé-Hoover, `taut = 50 fs` for Berendsen, and `friction = 0.1` (≈ 10 ps^{-1}) for Langevin unless otherwise mentioned. Other MD parameters were left to their default ASE values.

Bulk water model system. For this system, the NVT Nosé–Hoover thermostat and Langevin dynamics were compared. Target temperature was 300 K. The time step of 1 fs was considered small enough to properly resolve the vibrations with hydrogen mass set to the heavy value of 2.0 u. The simulations were extended to 20 ps (Langevin) and 18 ps (Nosé–Hoover) lengths. The Langevin friction coefficient was set to 0.02 (≈ 2 ps^{-1}), and Nosé–Hoover coupling time to 50 fs. The default density convergence criterion 10^{-4} e/v.e. was used which in practice led to $< 10^{-6}$ eV maximum total energy change. The van der Waals correction by Tkatchenko and Scheffler[10] was used for intermolecular long range interactions. Otherwise the DFT settings were the same as for N_2. In addition, we also performed microcanonical ensemble (constant NVE) simulations with velocity Verlet dynamics without thermostats to compare the results obtained with the above mentioned thermostats, and to further assess the effect of density convergence (10^{-4} vs 10^{-6} e/v.e.) on the DFT-MD performance.

Au(111)-H_2O system. Langevin dynamics (`friction = 0.1`) and Nosé–Hoover thermostat (`ttime = 50`) were also used for the Au(111)-H_2O system to control the temperature (set at 300 K and 350 K, respectively) in the simulations with 1 fs time step and 2 u mass for hydrogen atoms. This interface system was used to further test different SCF convergence setups. The trajectories compared in the main article were computed with a `eigenstates:1.0e-4, density:1.0e-5, energy:1e-6` setting. The density criterion determines the energy convergence, which was observed to be at least 10^{-7} eV. The same conditions were set to evaluate the effect of system-bath coupling parameter `ttime` (from 50 to 500 fs) in the Nosé–Hoover thermostatting. To explicitly test the density convergence (from 10^{-3} to 10^{-6} e/v.e.) the energy criterion was set sufficiently low.

Pt_{13}/ZrO_2 model system. Langevin thermalization at 300 K was used for the zero-temperature global minimum structure obtained before.[4] The Nosé–Hoover propagations were performed with two different convergence criteria overruling the GPAW defaults: a) 10^{-7} eV/v.e. for energy and b) 10^{-6} e/v.e. for density. In practice, the latter condition is tighter by several orders of magnitude. In addition, a Nosé–Hoover test run with GPAW defaults was propagated directly from the geometry-optimized structure, i.e., without thermalization to illustrate a severe case scenario of incorrect dynamics (see Section 3E). Otherwise the DFT settings with Tkatchenko–Scheffler vdW correction, and grid size were similar to the above Au-water case. Default ASE vdW parameters were used, except for Zr, for which no values are provided; instead, the Zr parameters were taken from Ref. 11.

Quantum ESPRESSO setup for N_2. We performed DFT-MD simulations using the Quantum ESPRESSO (QE) package[12] for the system of 64 N_2 molecules in a cubic box of length 30 Å. The Kohn-Sham states were expanded using a plane wave basis set with energy cut-offs of 30 Ry and 240 Ry for the kinetic energy and charge density, respectively. The interaction between the ionic core and valence electrons were treated with ultrasoft pseudopotentials[13] and the exchange-correlation interactions between the electrons were treated within the generalized gradient PBE approximation.[8] Brillouin zone sampling was done only at the zone center. The convergence threshold for self consistency was set to 10^{-9} Ry.

The velocity Verlet algorithm with 1 fs time step was used to integrate the equations of motions and the Berendsen thermostat was used with rise time parameter 1 fs to control the ionic temperature at 300 K. Thus, the Berendsen thermostat in practice reduced to the simple velocity rescaling thermostat.[9] After 10 ps run, the rise time was switched to 500 fs for another 5 ps. Initial coordinates were taken from the Langevin-thermalized ASE/GPAW run. Due to a mismatch in N_2 bond properties between the two software, the change led to excessive vibrational amplitude and energy in the beginning of the QE evaluation.

CP2K Setups. The CP2K calculations were performed using the CP2K/Quickstep code (version 6.1).[14] The core electrons were treated with norm-conserving GTH pseu-

dopotentials[15] while valence orbitals were expanded using the molecular-optimized double-ζ+polarization -type Gaussian basis functions.[16] The auxiliary plane-wave basis set cutoff was set to 400 Ry.

Unlike the other codes used in this work, CP2K includes a wide selection of advanced thermostats beyond common Berendsen and Nosé-Hoover algorithms. To facilitate fair comparison with the standard Nosé-Hoover thermostat as implemented in ASE and VASP, the Nosé-Hoover chain length in CP2K was set to one and the TEMP_KIND parameter was set as FALSE.

(CP2K) N_2 model system. The N_2 trajectories were simulated using the Langevin dynamics and the Nosé–Hoover thermostat to achieve the target temperature of 300 K. The Langevin dynamics were started from the pre-equilibratated GPAW/ASE structure while the Nosé–Hoover run was restarted from the CP2K Langevin run. A time step was set to 0.5 fs and the PBE functional was used.[8] The Langevin friction parameter and Nosé-Hoover time constant were set to 1.0 ps^{-1} and 50 fs, respectively.

(CP2K) Au(111)-H_2O model system. Each 7 ps CP2K trajectory was started from a pre-thermalized structure obtained from the Langevin dynamics ran with ASE/GPAW. The target temperature was set to 330 K. The SCF convergence was set to 10^{-6} Ha ($\approx 2.7 \times 10^{-5}$ eV). The Nosé–Hoover coupling time was 50 fs and Langevin friction was 1 ps^{-1}. The mass of 2.0 u was used for hydrogen to better facilitate the 1 fs time step. The PBE-D3 functional including C9 dispersion corrections[8,17] was used.

VASP setup for Au(111)-H_2O. The mass of hydrogen was set to normal 1.008 u. The self consistency criterion was set to 10^{-4} eV energy difference (EDIFF). Computationally very extensive trajectories were run for both Langevin and Nosé–Hoover temperature controls, totaling to about half a nanosecond.

DoS2PT analysis. The entropy calculations and kinetic energy partitioning analyses were performed using the DoS-2PT formalism[18,19] as implemented in the DoSPT code.[20] In DoS-2PT thermodynamic properties are computed from the system's density of states (DoS)

function using a two-phase thermodynamic (2PT) model. The theoretical development and computational methods are discussed in the original publications, and here we present only the minimal details needed for the kinetic energy partitioning.

The DoS is defined in terms of the mass-weighted summation of atomic spectral densities:

$$\mathrm{DoS}(\nu) = \frac{2}{k_\mathrm{B}T} \sum_{j=1}^{N} \sum_{k=1}^{3} m_j \, d_j^k(\nu) \,, \tag{1}$$

where N is the total number of atoms and m_j is the mass of atom j. The atomic spectral densities d_j^k are obtained from the DFT-MD computed velocity components v_j^k by using the Fourier transform of the velocity autocorrelation function $v(t)$,

$$d_j^k(\nu) = \frac{1}{\tau} \left| \int_0^\tau v_j^k(t) \mathrm{e}^{-i2\pi\nu t} \, \mathrm{d}t \right|^2 \,, \tag{2}$$

where τ is the total sampling time. For molecular species, the molecular spectral densities D_λ^k are used,

$$D_\lambda^k(\nu) = \sum_{j\in\lambda} m_j \frac{1}{\tau_\mathrm{a} - \tau_\mathrm{c}} \left| \int_{\tau_\mathrm{c}}^{\tau_\mathrm{a}} v_j^k(t) \mathrm{e}^{-\mathrm{i}2\pi\nu t} \, \mathrm{d}t \right|^2 \,, \tag{3}$$

where atoms j belong to the molecule λ. As molecules can break during a DFT-MD run, the sampling is weighted by the time intervals $\tau_\mathrm{a} - \tau_\mathrm{c}$ where a given molecule remains intact. Once the DoS has been computed, the entropy can be computed as an integral over the weighted DoS

$$S \propto k_\mathrm{B} \int_0^\infty \mathrm{DoS}(\nu) \, W(\nu) \, \mathrm{d}\nu \,, \tag{4}$$

where the weighting function $W(\nu)$ is computed within the 2PT formalism as detailed in Refs. 18–20.

To study the kinetic energy contribution from different degrees of freedom, the total DoS can be further partitioned into translational, rotational, and vibrational contributions as

$$\mathrm{DoS}(\nu) = \mathrm{DoS}_\mathrm{tr}(\nu) + \mathrm{DoS}_\mathrm{rot}(\nu) + \mathrm{DoS}_\mathrm{vib}(\nu) \,. \tag{5}$$

In practice, each DoS component is computed by separating the atomic/molecular velocity autocorrelation functions to the respective components

$$v_j^k(t) = v_{j,\mathrm{tr}}^k(t) + v_{j,\mathrm{rot}}^k(t) + v_{j,\mathrm{vib}}^k(t)\,. \tag{6}$$

The translational velocity of an atom/molecule is computed from its center of mass velocity. The rotational velocity is computed as $\overrightarrow{v}_j = \overrightarrow{\omega} \times \overrightarrow{r}_j$ where $\overrightarrow{r}_j$ is the position vector and the angular velocity $\overrightarrow{\omega}$ is obtained from the angular momentum $\overrightarrow{L}$ and inertia tensor $\mathbf{I}$ as $\overrightarrow{L} = \mathbf{I}\overrightarrow{\omega}$. The vibrational part is then obtained from Eq. (6). The effective temperature of each degree of freedom can be computed from the translation and rotational velocities using either the atomic/molecular mass or the moment of inertia, respectively. The vibrational temperature is obtained from the total kinetic energy after removing the rotational and translation contributions.[19,20] The integral over the DoS gives the total number of degrees of freedom (DoF) in the system

$$\int_0^\infty \mathrm{DoS}(\nu)\ \mathrm{d}\nu = 3N\,. \tag{7}$$

To obtain the number of DoF belonging to translation, rotation, or vibration, the total DoS in Eq. (7) is replaced with corresponding motion of the partitioned DoS function in Eq. (5).

The velocity autocorrelation functions $v(t)$ were obtained either by using velocities directly from trajectory if available (GPAW, CP2K), or by estimating velocities from the coordinates (VASP, QE). Other DoSPT options used were (i) the smoothing option (LOWESS filtering) to clarify the DoS plots, (ii) the vacuum option to handle the interfacial systems, and (iii) the excluding frozen volumes option to separate moving atoms from the immobile support layer. The kinetic energy partitioning to translational, rotational, and vibrational contributions can be found in Ref. 9 for the case N_2. This energy partitioning scheme for diatomic molecules was used to double check the results given by the DoSPT program. We note that the friction applied in the Langevin dynamics is rather small and it is not expected

to significantly influence the accuracy of the computed velocity autocorrelation functions.[21]

N_2 energy partitioning by ASE/GPAW, CP2K, and QE. For the ASE/GPAW simulations, the results from energy partition analyses are presented in Fig. S3. The trajectories begin with the correct energy partitioning that was obtained by Langevin thermalization. Both Berendsen and Nosé–Hoover thermostats quickly drained off the vibrations from the trajectories while translations and rotations showed a correlated variation and maintained the total kinetic energy close to 300 K. Both thermostats resulted in excess rotational energy (averaging at ca. 420 K) to compensate the lost vibrational intensity.

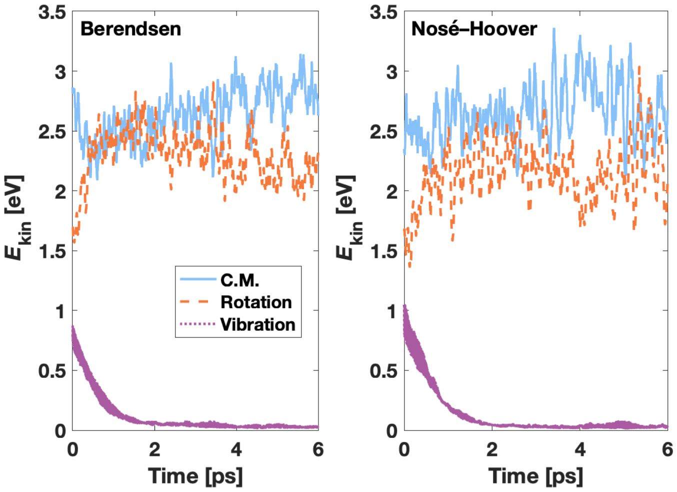

Figure S3: Kinetic energy decomposition to translational, rotational, and vibrational degrees of freedom for the N_2-system simulated by ASE/GPAW with 10^{-4} e/v.e. density convergence.

Better behavior is presented in Fig. S4 where the Langevin equilibration (last 13.5 ps) and tightly converged (10^{-6} e/v.e.) Nosé–Hoover are shown. Here, the temperatures averaged to 293 K (295 K C.M., 309 K rot., 256 K vib.) and 298 K (288 K, 300 K, 322 K), respectively. The partitioning was acceptable, except that Langevin lacks some vibrational energy.

The CP2K simulation results for N_2 are plotted in Fig. S5 using 50 fs moving averages to reduce the high-frequency vibrational scatter. The Langevin equilibration run, as started from the GPAW-biased structure, produced some drift in the rotational and vibrational energies. This stage also exhibited significant variations in oscillation amplitudes of the translational and rotational energies but overall the behavior is expected and correct. The

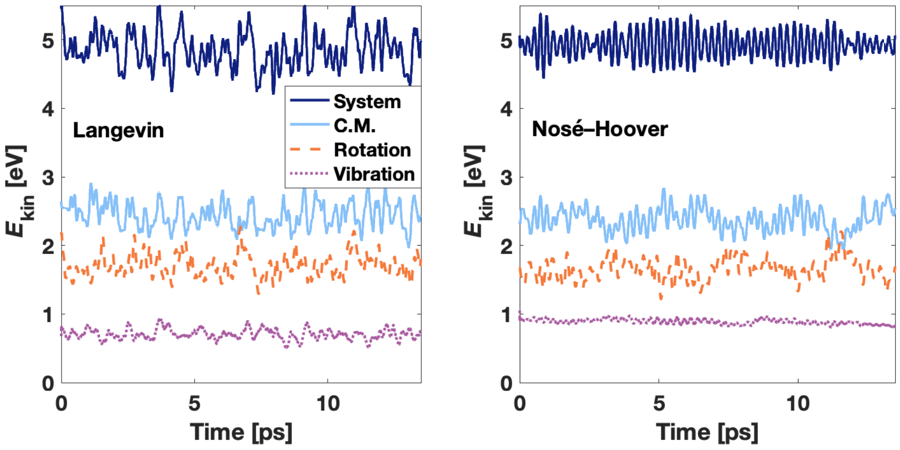

Figure S4: Kinetic energy decomposition to translational, rotational, and vibrational degrees of freedom for the N_2 system simulated by ASE/GPAW with Langevin and NH 10^{-6} e/v.e. density convergence. The curves are smoothed 100 fs moving averages.

subsequent Nosé–Hoover run maintained the average energy levels except for an upward drift of the rotational energy. The oscillation amplitudes were minor. From this plot one can not identify a significant flying ice cube effect. However, the kinetic energies clearly exhibited a synchronized, driven-like oscillation due to the thermostat. The in-phase nature of the periodicity (ca. 230 fs) is better visible in the temperature plot. This is artificial and should not exist. Such a correlation was absent in the ASE/GPAW Nosé–Hoover case above. The temperatures were fairly well in control in both runs. Langevin averaged to 322 K (318 K C.M., 327 K rot., 324 K vib.) and Nosé–Hoover to 327 K (332 K, 308 K, 352 K).

For the QE results, the last 5 ps of simple velocity rescaling simulation (rise time equals the time step) is presented in Fig. S6 together with the subsequent Berendsen simulation (rise time increased to 500 fs). The QE trajectory with aggressive, simple velocity rescaling exhibits a well-controlled average temperature of 298 K for the shown 5 ps period. The corresponding total kinetic energy thus appears fairly constant in time. However, the kinetic energy partitioning reveals 267 K, 303 K, and 378 K average temperatures for the center-of-mass, rotational, and vibrational degrees of freedom, respectively. As seen in the left-hand-side graph of Fig. S6, the C.M. and rotational motions seem highly correlated with each other while the high-frequency vibration appears more independent. The vibrational

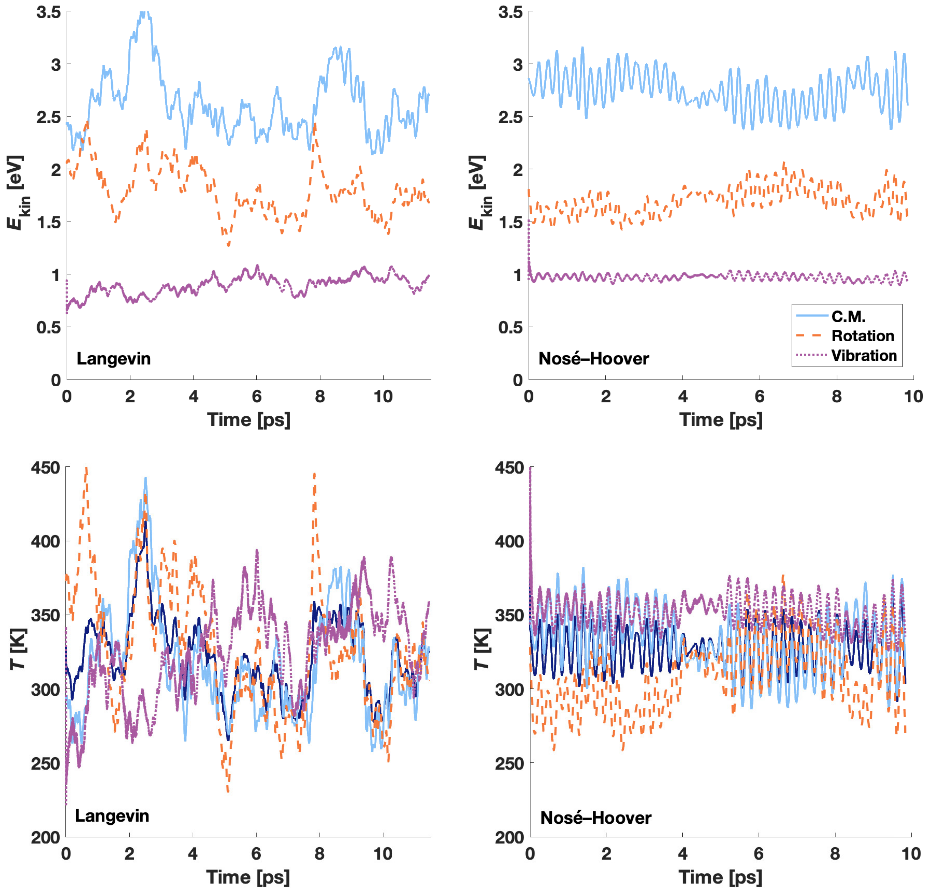

Figure S5: Kinetic energy partitions for the N_2 system thermostatted first by Langevin (left) and then by Nosé–Hoover (right) at 300 K. The DFT-MD trajectories were obtained using the CP2K code. Moving averages of trailing 50 fs (100 points) are plotted for C.M. translations, rotations and vibrations. The data is repeated on the bottom panels to plot the corresponding temperatures including also the total system temperature (solid, dark blue curves).

energy exhibits a slow damping in both magnitude and its oscillation amplitude; however, vibrations remain overheated during the present simulation time. This is in contrast to the behavior in ASE/GPAW trajectories, where vibrations were minor and quickly drained, see Figs. S3 and 1. We trace the origin for the overheated vibrational DoF to the initial distribution of N–N bond distances. The configuration from GPAW was not quite optimal for the QE procedure, and simulation time was not enough to get rid of the extra energy even with the aggressive coupling to thermostat. Despite the unfortunate bias, it seems clear that QE Berendsen contains a flying ice cube effect in a similar manner as in ASE/GPAW. The

simulation was continued with a thermostat rise time of 500 fs, shown in Fig. S6 on the right. The energy curves show a discontinuity from the left to right image due to restarting of the velocities. The less aggressive coupling results in more variation of the system temperature which again averages to 298 K. While the translations reside close to the target energy, suppressed rotations and overheated vibrations clearly deviate from equipartition. The overall vibrational energy remains constant in average but exhibits a marked beating pattern resembling artificial quantum correlation.

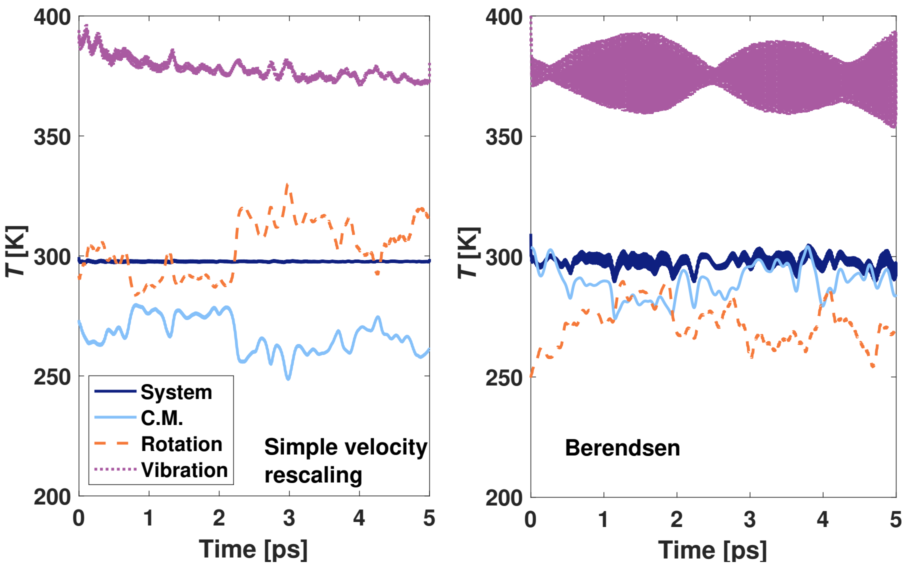

Figure S6: Kinetic energy partitioning given as temperature of translational, rotational, and vibrational degrees of freedom for the Berendsen-thermostatted N_2 system calculated by QE. Moving averages from trailing 10 fs periods were used to plot the data.

The DoSPT information on the degrees of freedom and the N_2 system entropies discussed above is collected in Table S1.

GPAW results for bulk water. The Nosé–Hoover thermostatted 18 ps run performed by ASE/GPAW is presented in Fig. S7. We observe that the temperature maintains within ±50 K bounds; however, rotations are overheated (average 449 K) with the expense of translation (239 K) and vibration (208 K). In contrast to bulk N_2, the vibrations are not damped down in this system. This short evaluation already demonstrates the unpredictability of the kinetic energy distribution when using the Nosé–Hoover thermostat. The effect is not in the

Table S1: N_2 entropies and numbers of DoF obtained by the three DFT-MD codes and different thermostats. The expected numbers are given in parentheses for center of mass, rotation, and vibration

Method	Thermostat	S (J K^{-1} mol^{-1})	DoF C.M.(192)	DoF Rot.(128)	DoF Vib.(64)
GPAW	Langevin[(a)]	86	190	133	54
	Nosé–Hoover 10^{-6}	123	185	129	69
	Nosé–Hoover 10^{-4}	153	201	179	5
	Berendsen	160	206	183	4
CP2K	Langevin	156	203	139	69
	Nosé–Hoover	173	214	137	75
QE	Berendsen	147	196	128	80

[(a)] full 18.5 ps trajectory, which includes the initialization stage

strict flying ice cube category as the lowest-frequency mode is under-represented. Similar variability in the kinetic energy drainage has been observed in a classical MD evaluation of a bulk water system,[22] where the effect depended on the time step and exhibited the opposite direction from low to high frequencies. Equipartition is fulfilled by the Langevin integrator, see the right panel in Fig. S7. Starting the 20 ps trajectory from a biased NPT (Nosé–Hoover) snapshot, the energies become corrected to proper distribution during the first 0.5 ps and vary within ±50 K amplitude. The 20 ps average temperatures are 290 K (total), 294 K (C.M.), 307 K (rot.), and 269 K (vib.).

The density of states analysis is presented in Fig. S8 for the bulk water system of 192 atoms. Langevin performs rather well in producing 188, 197 and 172 degrees of freedom (DoF) in C.M., rotational, and vibrational modes, respectively. With Nosé–Hoover the corresponding numbers are clearly off at 153, 287, and 133 indicating that the number of DoF corresponding to these modes are incorrectly reproduced by Nosé–Hoover. In this case, the flying ice cube effect manifests itself in the energy transfer from the highest-frequency mode (70 ps^{-1}) to lower-frequency rotation, but not to translation. The entropy values are less sensitive to the difference in number of DoF and turn out as 40 J $K^{-1}mol^{-1}$ by the Langevin trajectory and 44 J $K^{-1}mol^{-1}$ by the Nosé–Hoover, both markedly below the experimental water value of 70 J $K^{-1}mol^{-1}$. The vibrational frequencies reflect the properties of the vdW-

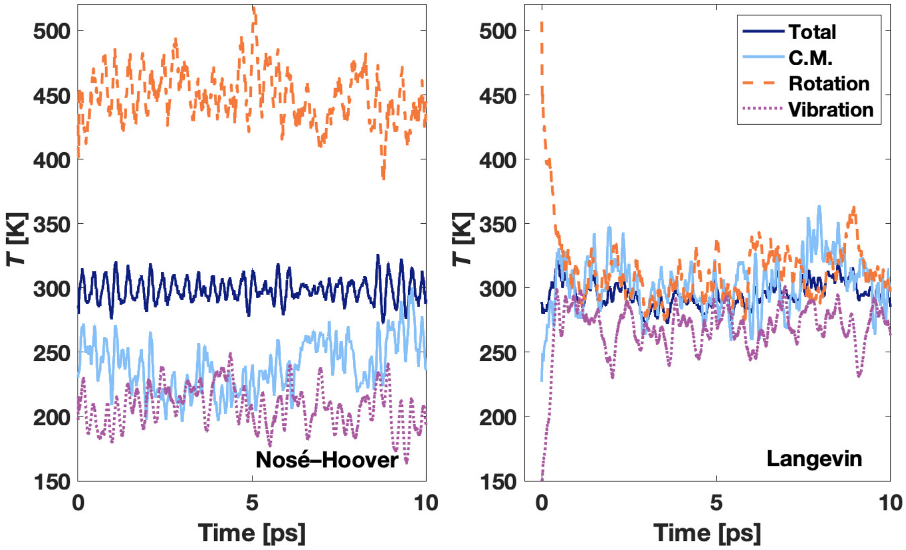

Figure S7: Kinetic energy partitioning in the bulk water system calculated by GPAW with Nosé–Hoover (left) and Langevin (right) settings for bath coupling. For clarity, the temperatures are plotted using moving averages of trailing 100 fs, and only the first 10 ps are shown.

corrected PBE-TS09 functional. These numbers are intact by the integrator method. The peaks at 35 ps^{-1} (bend) and 70 ps^{-1} (stretch) are red-shifted from the experimental values 40 and 75 ps^{-1} (scaled by $\sqrt{(2)}$ for heavy water).

In NVE dynamics, using the default 10^{-4} e/v.e. convergence fails to provide constant energy or energy conservation; instead, a leakage of ca. 0.2 eV/ps is observed and consequently the number of degrees of freedom drop below 192 for each mode. The energy is unevenly partitioned and show diminished vibrations, see Fig. S9a). Markedly different result is obtained with tighter 10^{-6} e/v.e. convergence as shown in Fig. S9b). The total energy is conserved and the partitioning is correct with numbers of DoF at 193, 192, and 188. In particular, the temperature is in such a proper control for this system that it raises a question about the need of thermostatting in the first place. The total temperature maintains at 300 K and its histogram has a full width at half maximum of 30 K only. The entropy evaluation yields 49 J K^{-1} mol^{-1} which is higher than what Langevin produced and closer to the experimental value.

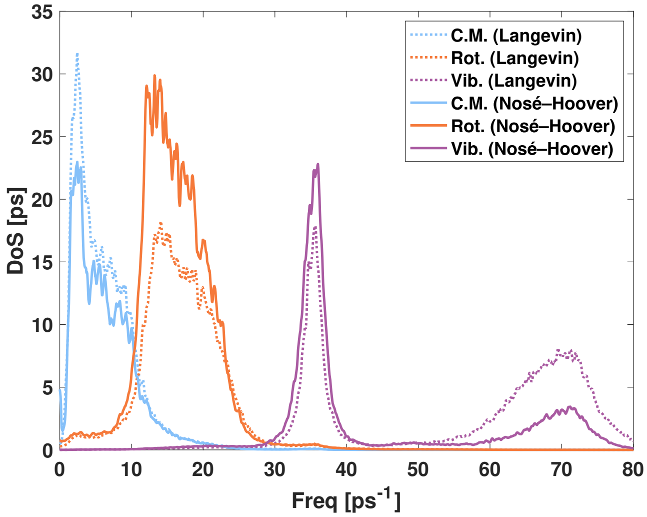

Figure S8: Density of states plot for the bulk (heavy) water system calculated by GPAW with Langevin dynamics (dotted curves) or Nosé–Hoover thermostat (solid).

The density of states results, discussed above for the bulk water system and below for the water–metal interface system, are compared in Table S2. The experimental standard entropy is 70 J K^{-1} mol^{-1} for bulk water[23] and 47 J K^{-1} mol^{-1} for Au.[24] As outlined in the main article, VASP performs correctly with the Langevin dynamics and produces the right numbers of DoF, 96 for H_2O and 72 for Au. The use of Nosé–Hoover thermostat results in excess translational DoF and the entropies are correspondingly higher. The DoF from the GPAW–Langevin simulation are close to those obtained with VASP. The resulting entropy values are lower than with VASP which reveals the effect of the larger hydrogen masses used and thus the different frequencies weighted in Eq. 4. With the CP2K setup, both the Langevin and Nosé–Hoover results indicate that the 7 ps simulation time was insufficient for reliable thermodynamic evaluation.

GPAW results for Au(111)-water interface. We illustrate the behavior of the Nosé–Hoover thermostat for the Au(111)-H_2O system by showing the impact of density or energy convergence in Fig. S10. The top left panel contains results from the below-default settings 10^{-3} e/v.e., 10^{-3} eV/v.e. In this case, density convergence was the decisive criterion and energy was converged to at least 10^{-2} eV. The top right panel contains tightened energy condition (10^{-4} eV/v.e.) which yielded at least 5×10^{-3} eV convergence in practice; however,

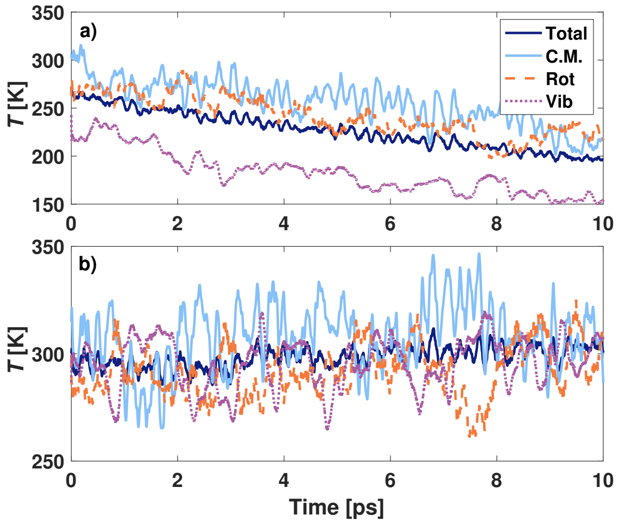

Figure S9: Comparison of energy partitioning for bulk water obtained by NVE simulations with a) 10^{-4} e/v.e. and b) 10^{-6} e/v.e. convergence criteria. Last 10 ps are presented using 100 fs moving averages.

with marginal impact on performance. Then, in the lower left panel, the convergence criterion was further tightened to 10^{-5} eV/v.e. which produced at least 10^{-3} eV accuracy. These panels show that the error in kinetic energy partitioning is aggravated with loose convergence criteria. This is most clearly seen in curves that represent the translational energy of the Au atoms. The Au surface cooled down below 100 K within few picoseconds as energy was transferred to heat up the H and O atoms. Only the most tight convergence case (bottom right) appears reasonable as the atomic constituents were not strayed too far from the full system average. This panel contains seemingly well behaving data from 10^{-5} e/v.e., 10^{-6} eV/v.e. setting, where density was once more the ruling factor, yielding less than 10^{-7} eV total energy change for self consistent iterations.

We used the tight convergence criteria (10^{-5} e/v.e., 10^{-6} eV/v.e.) to examine the impact of the coupling timescale parameter to the Nosé–Hoover performance. The result is illustrated in Fig. S11. Increasing the coupling efficiency by reducing the characteristic time from 500 fs to 50 fs obviously promotes the settling to equilibrium, and it is also shown to squeeze the distribution of atomic energies closer to the system average.

To judge whether the Nosé–Hoover realization of the temperature control is acceptable

Table S2: H_2O [and Au] entropies and number of DoF obtained from the simulations of bulk water and water–Au interface

System	Code	Method	S (J $K^{-1}mol^{-1}$)	DoF		
				CM	Rot	Vib
Bulk	GPAW	Langevin	40	188	197	172
		Nosé–Hoover	44	153	287	133
		NVE	49	193	192	188
Interface	VASP	Langevin	50 [48]	96 [72]	96	96
		Nosé–Hoover	61 [63]	101 [82]	96	83
	GPAW	Langevin	37 [32]	95 [70]	96	92
		Nosé–Hoover	66 [54]	105 [74]	108	82
	CP2K	Langevin	74 [59]	106 [74]	103	111
		Nosé–Hoover	60 [41]	92 [50]	88	127

at 10^{-5} e/v.e. convergence, we compare it to the Langevin dynamics of the same system. To this end, we present the kinetic energy partitioning in Fig. S12. Although the atom-specific energies seem well controlled, the resolution to translational, rotational, and vibrational degrees of freedom again reveal the flying ice cube problem. Vibrations are clearly under-represented in the Nosé–Hoover case at least during the examined period. Langevin simulation is genuinely maintained at 300 K and obey the correct equipartition all the time.

To have an additional viewpoint into the Nosé–Hoover imbalance, the DoS corresponding to above simulations is presented in Fig. S13. In this plot, the striking difference is present at low-frequency modes of both H_2O and Au. It is clear that the simulation time is too short to yield proper sampling of the phase space but the method differences are visible. The water DoF sum up to 105 translations, 108 rotations, and 82 vibrations. For Langevin dynamics, the numbers are much closer to 96 (95, 96, and 92, respectively). It can be seen in the atom-specific energy plots in Table 1 that the simulations had no trouble in producing a thermalized Au surface. For Au, both methods reach close to the proper 72 translations, 74 by Nosé–Hoover and 70 by Langevin, but the former has a suspiciously peaked DoS structure.

CP2K results for Au(111)-water interface. Fig. S14 shows the kinetic energy parti-

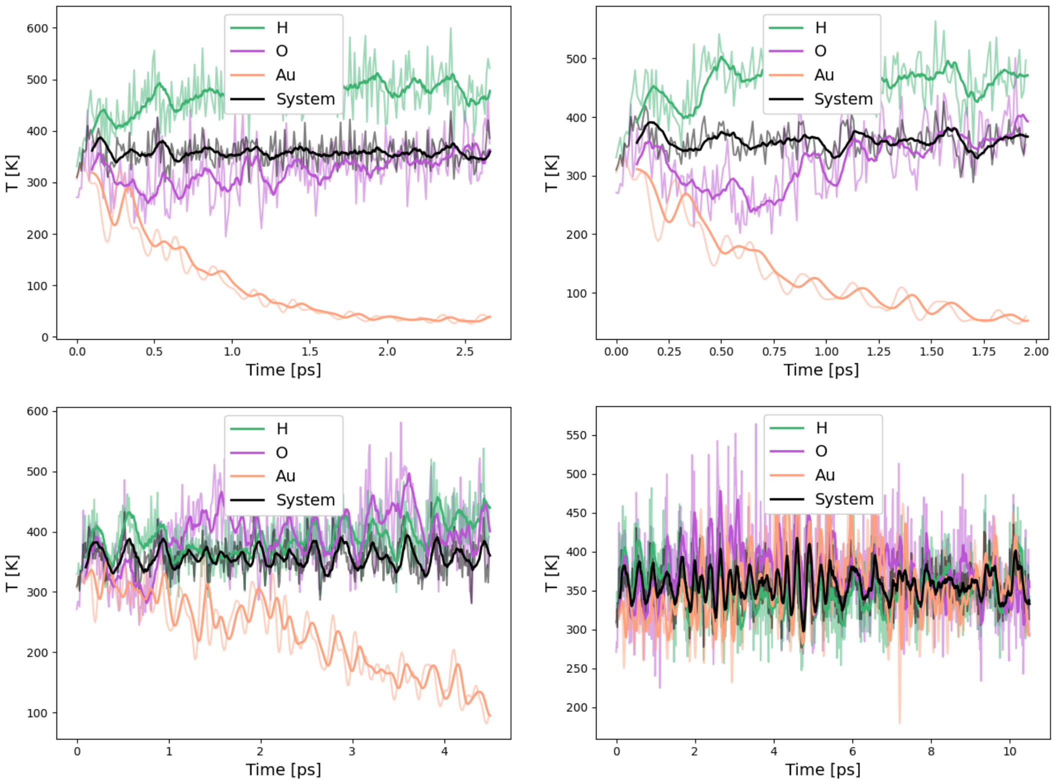

Figure S10: Effect of energy convergence on the temperature distribution in GPAW Au(111)-H_2O system from Nosé–Hoover thermostatting aimed at 350 K. From left to right, the top row panels correspond to energy convergence of 10^{-3} and 10^{-4} eV/v.e., and the bottom row to 10^{-5} and 10^{-6} eV/v.e. The density convergence was 10^{-3} e/v.e for all but the bottom right panel that replicates the data from Table 1. The light trace is every 10th step and the thick line 0.1 ps moving average.

tioning into center of mass (C.M.), rotational, and vibrational degrees of freedom as present in the 7 ps trajectories controlled by the Langevin dynamics or the Nosé–Hoover thermostat. In this comparison, the total kinetic energy appears to drift more in the Langevin than in the Nosé–Hoover case. During this short simulation period the Langevin temperature averages as high as to 359 K, and the C.M., rotational, and vibrational degrees of freedom all follow the system temperature behavior (349 K, 353 K, and 381 K, respectively).

With the Nosé–Hoover, the total kinetic energy and temperature are fairly well in control as customary. However, the H_2O vibrations appear overheated, and after 5 ps they start to drift markedly from the total average of 326 K. This happens more notably at the expense of translational DoF that involves the gold surface. Looking at the atom-specific energies

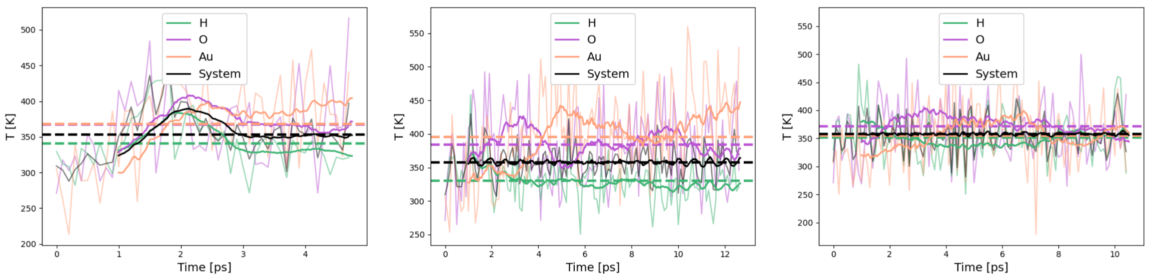

Figure S11: Effect of the Nosé–Hoover coupling time constant on the temperature distribution in Au(111)-H_2O: From left to right the timescale parameter is 500, 100, and 50 fs. The thin lines are every 100th data point, the dashed lines are averages, and thick lines are moving averages with 0.5 ps trailing.

shown in Table 1 of the main article, it can be seen that overheated vibrations correspond to high-temperature hydrogen atoms. It is intriguing to see that there is energy transfer from surface to liquid and internal re-partitioning within the water molecules.

Fig. S15 shows the density of states for the center of mass, rotational, and vibrational degrees of freedom of water, and translations of the Au atoms on the surface, with the aim at comparing Langevin and Nosé–Hoover methods implemented in the CP2K program. The DoS curves are surprisingly similar in shape. The peak maxima appear at same frequencies, and only intensity level differences are notable. Integrating the intensity curves, the methods should yield 72 DoF for Au and 96 DoF for each water mode. For Au, the number of DoF is 74 by the Langevin dynamics and only 50 by the Nosé–Hoover. For water, the numbers are (106, 103, 111) by Langevin and (92, 88, 127) by Nosé–Hoover. That is, the numbers indicate that some of the energy becomes transferred from Au translations to H_2O vibrations in the case of CP2K/Nosé–Hoover. The discrepancy even in the supposedly well-behaving Langevin case reveals the insufficiency of the present simulation length at 7 ps to yield meaningful thermodynamics analysis. However, the DoS are valuable to illustrate differences in kinetic energy partitioning. By Langevin, the entropy values turn out as 59 J K^{-1} mol^{-1} and 74 J K^{-1} mol^{-1} for Au and water, respectively. With Nosé–Hoover, these values are 41 J K^{-1} mol^{-1} and 60 J K^{-1} mol^{-1}. Again, clear method differences are found.

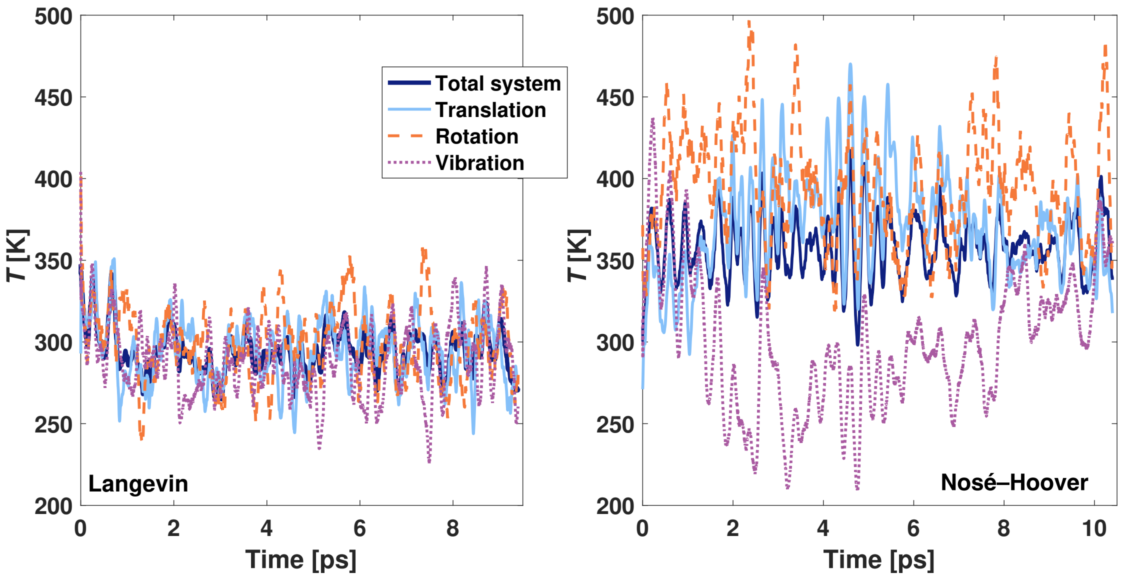

Figure S12: partitioning of kinetic energy in the Au(111)-H_2O system calculated by ASE/GPAW with 10^{-5} e/v.e. density convergence. Langevin dynamics targeted at 300 K and Nosé–Hoover thermostatted at 350 K. The curves are plotted as 0.1 ps moving averages to reduce scatter.

GPAW results for Pt_{13}/ZrO_2. In the main article, we notice that the temperature of the supported cluster is erroneously described by the Nosé–Hoover thermostat under the studied conditions. To further demonstrate the incorrectness, we took under study a deliberately dubious case where Nosé–Hoover 300 K simulation was started directly from the 0 K minimum structure (a Nosé–Hoover run should always be pre-thermalized).[25] Here, an especially severe effect is observed: the temperature of the cluster initially peaks at over 5000 K and remains around 1000 K, while the support cools down to ca. 200 K (Fig. S16). The temperature of the cluster is thus considerably higher than the intended 300 K, which causes its mobility and flexibility to be overestimated. Again, we stress that the average temperature of the system settles at about 300 K as expected, but the temperature distribution is completely wrong in this case. However, the Nosé–Hoover controlled temperatures may be incorrect even when the structures have been properly equilibrated with a Langevin run.

GPAW results for Cu(100/111)-H_2O (earlier study). We analyzed recent DFT-

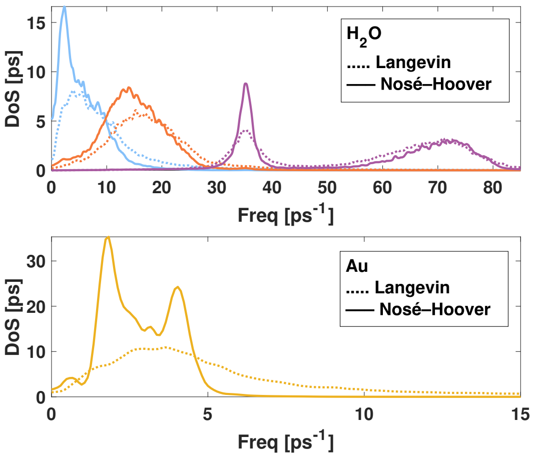

Figure S13: DoS for Au(111)-H_2O system from the ASE/GPAW Langevin dynamics at 300 K (dotted curves) and Nosé–Hoover (solid) trajectories at 350 K.

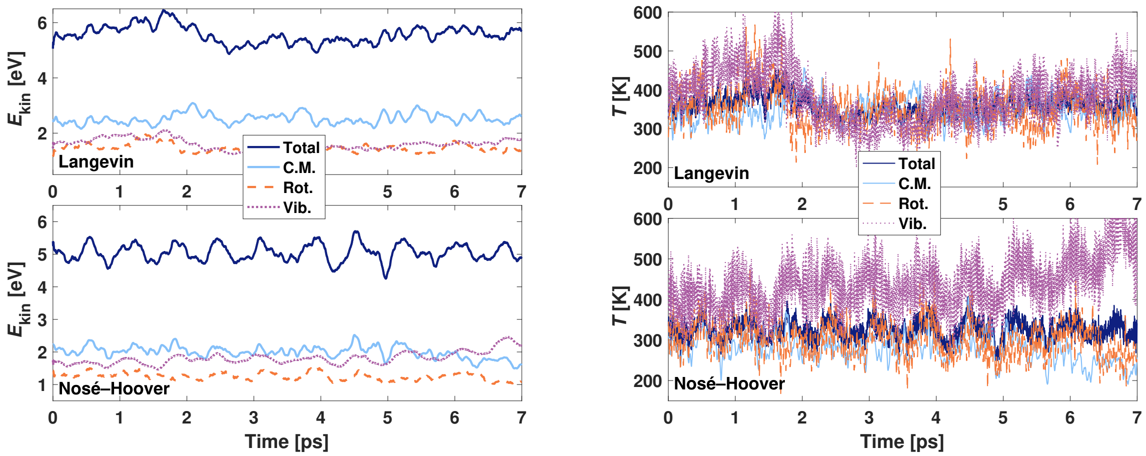

Figure S14: Kinetic energy (presented as temperature on the right) partitioning for the CP2K simulations of the Au(111)-H_2O system with Langevin dynamics or Nosé–Hoover thermostat. The energies are smoothed for clarity by 0.1 ps moving averages, while temperatures show the full extent of variation.

MD trajectories from literature[1] to obtain atom-specific temperatures in systems of solvated and hydrogenated Cu(100) and Cu(111). The referenced work utilized the Berendsen thermostat as implemented in ASE and the energies and forces were computed using GPAW in the LCAO mode with the energy convergence value of 10^{-3} eV. In these simulations all the Cu atoms and eight water molecules were held fixed while 24 water molecules and the adsorbed species were mobile. For further computational details, please refer to the original publication.

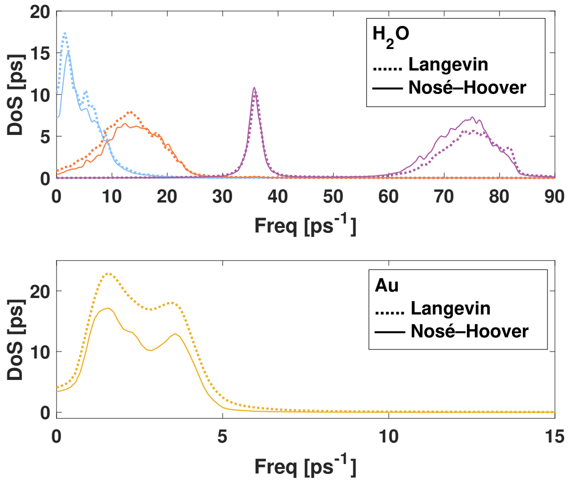

Figure S15: Density of states plots for the Au(111)-H_2O system obtained from 7 ps CP2K trajectories with Langevin (dashed curves) and Nosé–Hoover (solid) implementations.

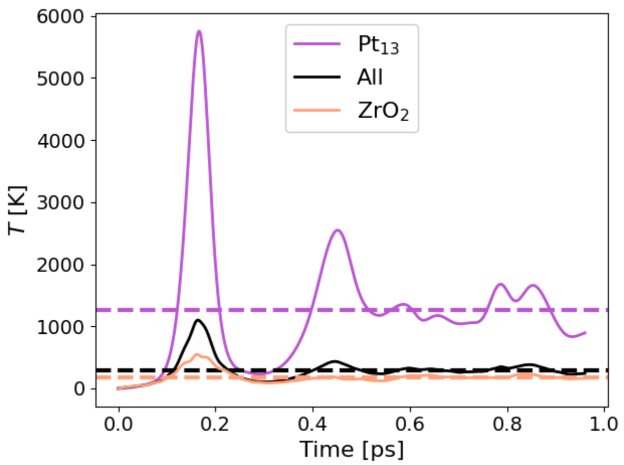

Figure S16: Temperatures of Pt_{13} cluster and ZrO_2 surface when Nosé–Hoover was started directly from the 0 K structure. No smoothing has been applied.

The results in Fig. S17 show that both pristine and hydrogen-covered, solvated copper surfaces have temperature gradients in the system. Rather than achieving the desired temperature of 300 K, the temperature converges to around 150 K. This discrepancy likely results from an error in handling the degrees of freedom that are removed when parts of the system are constrained. Besides this issue, on the pristine Cu(100) the oxygen and hydrogen temperatures of water differ by 50 K while on the Cu(111) both atom types have the same temperature. More noticeable differences are seen for the hydrogen-covered surfaces where the adsorbed atoms are several hundred degrees hotter initially.

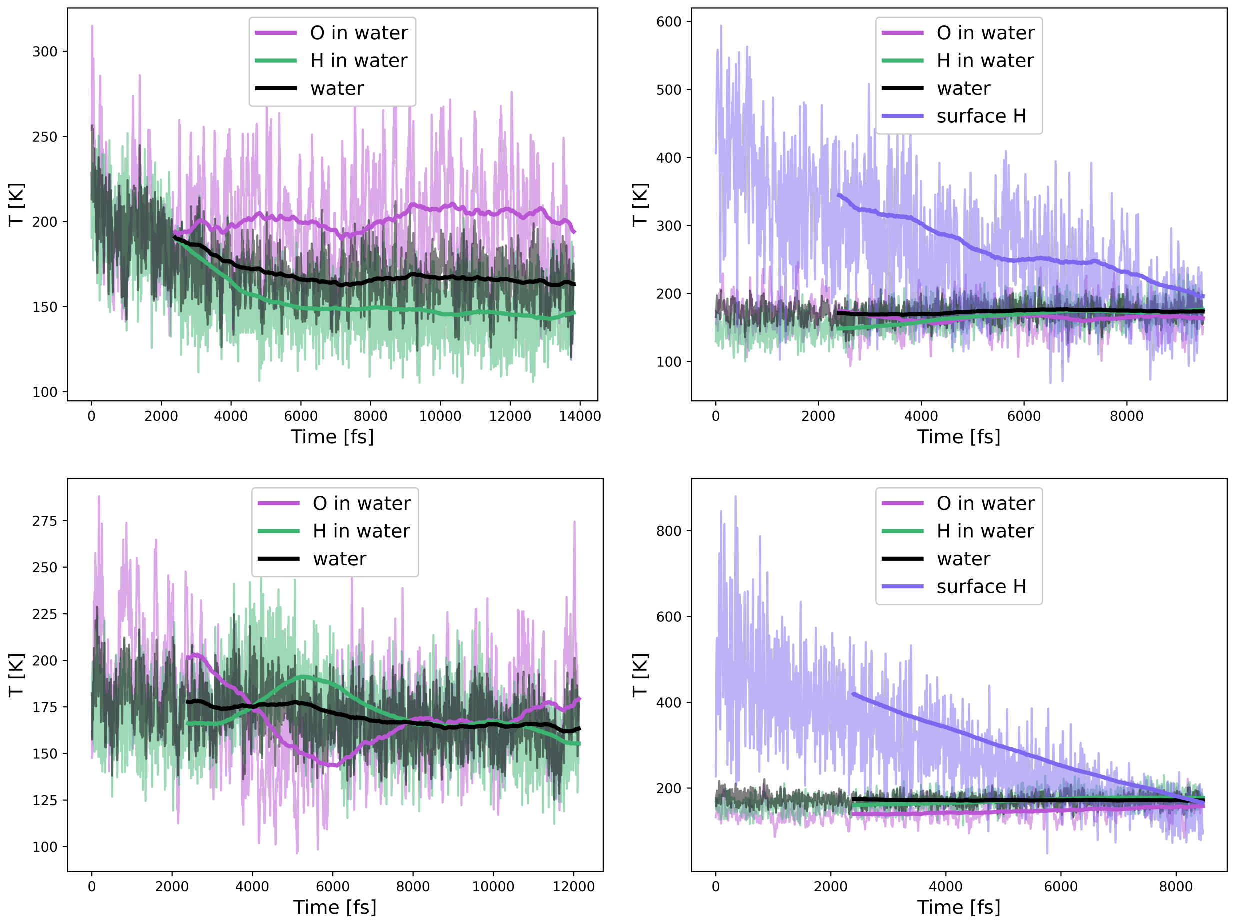

Figure S17: Temperatures extracted from the database trajectories provided by Ref. 1. Upper row: solvated Cu(100) and solvated Cu(100) with six H atoms on the surface. Lower row: solvated Cu(111) and solvated Cu(111) with six H atoms on the surfaces.

VASP results for Au(111)-H_2O-Li-CO_2 (earlier study). We reanalyzed some recent AIMD trajectories from a study on cation effects on CO_2 reduction.[2] Specifically, we studied the atom-specific temperatures of two systems: a solvated gold surface with two Li ions in the liquid with and without an adsorbed CO_2. The simulations were carried out using VASP with a 450 eV cut-off for the plane-wave basis but no convergence values were reported. The Nosé–Hoover thermostat was applied to keep the average temperature at 300 K but no other information was given.

The results in Fig. S18 show that the average temperature is controlled well and it remains very close to the target temperature throughout the short simulation. The atomic temperatures, however, exhibit very large variations in both systems. In particular, at the start of each simulation, the Li ions and the CO_2 molecule exhibit very high temperatures exceeding the target by several hundred Kelvins — this shows that despite the correct average temperature the system is not in equilibrium. After ~ 1 ps all atomic temperatures settle around the target temperature but the hydrogen and oxygen atoms of water are at different temperatures, Li ions exhibit significant fluctuations and different temperatures in the two simulated systems, and the CO_2 appears to be colder than the other atoms. This analysis clearly demonstrates that the models exhibit notable temperature differences between different atoms and that the simulated systems are far from a thermodynamic equilibrium. While we only analyzed two systems from the variety of different ones of Ref. 2, we expect that similar issues are present in the other studied systems as well since they are structurally very similar and the same computational setup was used for all the simulations. This discussion also pertains to another study[26] which addressed cation effects on a gold-catalyzed CO_2 electroreduction using an identical computational setup.

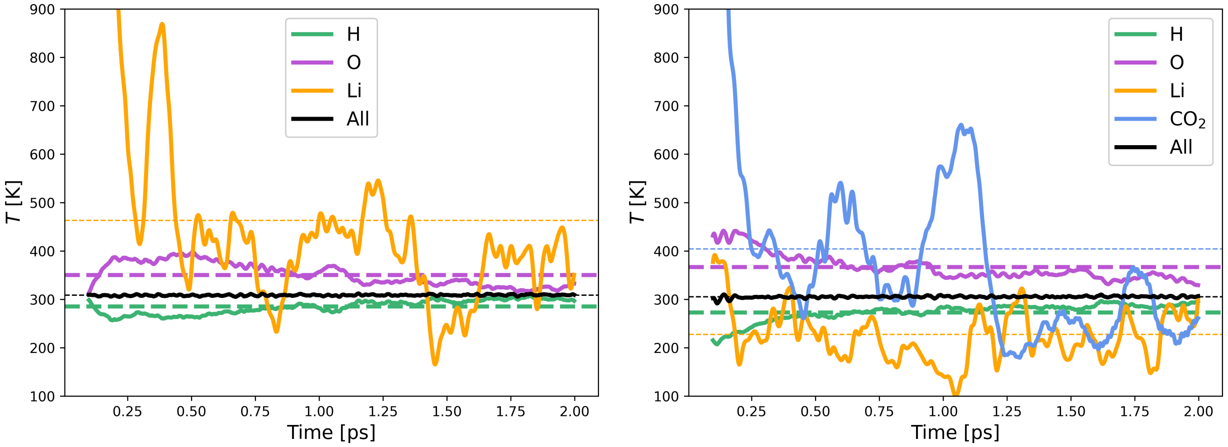

Figure S18: Temperatures extracted from the database trajectories provided by Ref. 2. Upper row: solvated Au(111) with two Li ions, and solvated Au(111) with two Li ions and an adsorbed CO_2

References

(1) Sebastián-Pascual, P.; Petersen, A. S.; Bagger, A.; Rossmeisl, J.; Escudero-Escribano, M. pH and Anion Effects on Cu–Phosphate Interfaces for CO Electroreduction. *ACS Catal.* **2021**, *11*, 1128–1135.

(2) Monteiro, M. C. O.; Dattila, F.; López, N.; Koper, M. T. M. The Role of Cation Acidity on the Competition between Hydrogen Evolution and CO_2 Reduction on Gold Electrodes. *J. Am. Chem. Soc.* **2022**, DOI:10.1021/jacs.1c10171.

(3) Le, J.; Cuesta, A.; Cheng, J. The structure of metal-water interface at the potential of zero charge from density functional theory-based molecular dynamics. *J. Electroanal. Chem.* **2018**, *819*, 87 – 94.

(4) Bazhenov, A. S.; Honkala, K. Globally Optimized Equilibrium Shapes of Zirconia-Supported Rh and Pt Nanoclusters: Insights into Site Assembly and Reactivity. *J. Phys. Chem. C* **2019**, *123*, 7209–7216.

(5) Mortensen, J. J.; Hansen, L. B.; Jacobsen, K. W. Real-space grid implementation of the projector augmented wave method. *Phys. Rev. B* **2005**, *71*, 035109.

(6) Enkovaara, J. et al. Electronic structure calculations with GPAW: a real-space implementation of the projector augmented-wave method. *J. Condens. Matter Phys.* **2010**, *22*, 253202.

(7) Larsen, A. H. et al. The atomic simulation environment—a Python library for working with atoms. *J. Condens. Matter Phys.* **2017**, *29*, 273002.

(8) Perdew, J. P.; Burke, K.; Ernzerhof, M. Generalized Gradient Approximation Made Simple. *Phys. Rev. Lett.* **1996**, *77*, 3865.

(9) Braun, E.; Moosavi, S. M.; Smit, B. Anomalous Effects of Velocity Rescaling Algorithms: The Flying Ice Cube Effect Revisited. *J. Chem. Theory Comput.* **2018**, *14*, 5262–5272.

(10) Tkatchenko, A.; Scheffler, M. Accurate Molecular Van Der Waals Interactions from Ground-State Electron Density and Free-Atom Reference Data. *Phys. Rev. Lett.* **2009**, *102*, 073005.

(11) Gould, T.; Bučko, T. C6 Coefficients and Dipole Polarizabilities for All Atoms and Many Ions in Rows 1–6 of the Periodic Table. *Journal of Chemical Theory and Computation* **2016**, *12*, 3603–3613, PMID: 27304856.

(12) Giannozzi, P. et al. QUANTUM ESPRESSO: A Modular and Open-source Software Project for Quantum Simulations of Materials. *J. Phys.: Condens. Matter* **2009**, *21*, 395502.

(13) Vanderbilt, D. Soft Self-Consistent Pseudopotentials in a Generalized Eigenvalue Formalism. *Phys. Rev. B* **1990**, *41*, 7892.

(14) Kühne, T. D. et al. CP2K: An electronic structure and molecular dynamics software package - Quickstep: Efficient and accurate electronic structure calculations. *J. Chem. Phys.* **2020**, *152*, 194103.

(15) Goedecker, S.; Teter, M.; Hutter, J. Separable dual-space Gaussian pseudopotentials. *Phys. Rev. B* **1996**, *54*, 1703–1710.

(16) VandeVondele, J.; Hutter, J. Gaussian basis sets for accurate calculations on molecular systems in gas and condensed phases. *J. Chem. Phys.* **2007**, *127*, 114105.

(17) Grimme, S.; Antony, J.; Ehrlich, S.; Krieg, H. A consistent and accurate ab initio parametrization of density functional dispersion correction (DFT-D) for the 94 elements H-Pu. *J. Chem. Phys.* **2010**, *132*, 154104.

(18) Lin, S.-T.; Blanco, M.; Goddard, W. A. The two-phase model for calculating thermodynamic properties of liquids from molecular dynamics: Validation for the phase diagram of Lennard-Jones fluids. *J. Chem. Phys.* **2003**, *119*, 11792–11805.

(19) Lin, S.-T.; Maiti, P. K.; Goddard, W. A. Two-Phase Thermodynamic Model for Efficient and Accurate Absolute Entropy of Water from Molecular Dynamics Simulations. *J. Phys. Chem. B* **2010**, *114*, 8191–8198.

(20) Caro, M. A.; Laurila, T.; Lopez-Acevedo, O. Accurate schemes for calculation of thermodynamic properties of liquid mixtures from molecular dynamics simulations. *J. Chem. Phys.* **2016**, *145*, 244504.

(21) Basconi, J. E.; Shirts, M. R. Effects of Temperature Control Algorithms on Transport Properties and Kinetics in Molecular Dynamics Simulations. *J. Chem. Theory Comput.* **2013**, *9*, 2887–2899.

(22) Yan, L.-M.; Sun, C.; Liu, H.-T. Opposite phenomenon to the flying ice cube in molecular dynamics simulations of flexible TIP3P water. *Adv. Manuf.* **2013**, *1*, 160–165.

(23) Chase, M. W., Jr. NIST-JANAF Thermochemical Tables, 4th Edition. *J. Phys. Chem. Ref. Data, Monograph 9* **1998**, 1–1951.

(24) Geballe, T. H.; Giauque, W. H. The Heat Capacity and Entropy of Gold from 15 to 300 °K. *J. Am. Chem. Soc.* **1952**, *74*, 2368–2369.

(25) Tuckerman, M. *Statistical Mechanics: Theory and Molecular Simulations*; Oxford University Press, 2010.

(26) Monteiro, M. C. O.; Dattila, F.; Hagedoorn, B.; García-Muelas, R.; López, N.; Koper, M. T. M. Absence of CO2 electroreduction on copper, gold and silver electrodes without metal cations in solution. *Nat. Catal.* **2021**, *4*, 654–662.
