# Supplementary material for: Addressing Dynamics at Catalytic Heterogeneous Interfaces with DFT-MD: Anomalous Temperature Distributions from Commonly Used Thermostats
Source: J Phys Chem Lett. 2022 Mar 17;13(11):2644–52. doi: 10.1021/acs.jpclett.2c00230 (PMC8959310; doi:10.1021/acs.jpclett.2c00230)
Supplement: Supplementary file 2 — jz2c00230_si_002.pdf [file jz2c00230_si_002.pdf]

jz-2022-00230c.R1

Name: Peer Review Information for "Addressing Dynamics at Catalytic Heterogeneous Interfaces With DFT-MD: Anomalous Temperature Distributions From Commonly Used Thermostats"

First Round of Reviewer Comments

Reviewer: 1

Comments to the Author

The paper reports that the most popular Nose–Hoover and Berendsen thermostats used in MD simulations in various periodic codes, produce the so-called "flying ice cube effect" whereby the temperature, while correctly conserved, is not correctly distributed over the degrees of freedom of the system and over parts of the system. A good representation of typical systems of interest are used to illustrate the problem (box of apolar molecules, solvated Au electrode, supported cluster catalyst). Langevin dynamics is recommended instead, to avoid the ice cube problem. The paper is technically very solid, and also very well written. I believe it is of great interest to theorist in the field of heterogeneous catalysis, where ab initio MD is used fairly often, and these simulations indeed lead to big conclusions often published in high-profile journals. This particular community needs to be aware of the issue, and I am in favour of publishing the paper. I am not sure about appealing to the broad p-chem readership of JPCLett, and would propose JCTC as the venue. However, in JPCLett the paper probably has a greater chance to be seen by those who need to read it. So I do not really oppose to publishing it here.

Reviewer: 2

Comments to the Author

This manuscript discussed the anomalous temperature distributions during the DFT-MD simulations of catalytic heterogeneous interfaces with the commonly used thermostats. The simulated temperatures were partitioned into translational, rotational, and vibrational kinetic energies. And it is concluded that the Nose-Hoover, Berendsen thermostats and the simple velocity-scaling failed to provide a reliable temperature distribution among these degrees of freedom (similar to the flying-ice-cube effect). Therefore, the manuscript recommended that the Nosé-Hoover and Berendsen thermostats should be used with care and the Langevin thermostat is always recommended.

Did the authors noticed a similar paper about the temperature distributions among the different degrees of freedom using classic MD simulation (Yan LM, et al. Adv. Manuf. 2013, 1(2) 160)? What were

the differences between these two papers: the simulated systems, the MD method, and the thermostats? What is the progress of the present manuscript compared with the pervious paper?

Author's Response to Peer Review Comments:

Dear Editor,

thank you for your email. I provide the separate cover letter, which contains the detailed response to the referees comments and explains the modification we hade made. We hope that the manuscript can be now accepted to the Journal of Physical Chemistry Letters.

Sincerely Yours,

Karoliina Honkala

Dear Editor,

Thank you for your email regarding our manuscript. We wish to thank the reviewers for their valuable work and hope that the updated manuscript will be found suitable for publication in The Journal of Physical Chemistry Letters.

We acknowledge the first reviewer's note regarding the publication venue but fully agree with their assessment that "in JPCLett the paper probably has a greater chance to be seen by those who need to read it".

Please note that the TOC figure of the manuscript has been updated.

Below we respond to the second reviewer's comments in detail. These comments have been included in black, our responses in blue, and modifications to the text in *italics*.

Sincerely, on behalf of all the authors,

Karoliina Honkala

Reviewer 2

a) Did the authors noticed a similar paper about the temperature distributions among the different degrees of freedom using classic MD simulation (Yan LM, et al. Adv. Manuf. 2013, 1(2) 160)? **b)** What were the differences between these two papers: the simulated systems, the MD method, and the thermostats? **c)** What is the progress of the present manuscript compared with the pervious paper?

a) We thank the referee for pointing out this paper, which focuses on classical molecular dynamics simulations for bulk water. We would like to point out that the flying ice cube effect is well known in classical MD literature, which was already extensively cited in our original manuscript. We now cite the Yan et al. paper in the main manuscript (see citation 68) and briefly discuss it in supporting information, where we present our results for bulk water.

The following text has been added to chapter 3B in SI:

This short evaluation already demonstrates the unpredictability of the kinetic energy distribution when using the Nosé–Hoover thermostat. The effect is not in the strict flying ice cube category as the lowest-frequency mode is under-represented. Similar variability in the kinetic energy drainage has been observed in a classical MD evaluation of a bulk water system [22] where the effect depended on the time step and exhibited the opposite direction from low to high frequencies.

b) As an answer to referee's question how our work differs from the Yan et al. 2013 work, we point out the following:

- Yan et al. focused on classical MD whereas we discuss DFT-MD, which has its own considerations, especially convergence issues.
- We discuss multiple thermostats and demonstrate the flying ice cube effect over multiple DFT-MD implementations, while Yan et al. only used one software and the Nosé–Hoover thermostat.
- We extend the discussion from homogeneous fluids to heterogeneous or two-component systems, demonstrating the possible issues for several systems of current interest instead of only looking at simple model systems like bulk water.
- Finally and most importantly, we highlight that the flying ice cube effect is not just a minor theoretical detail for MD experts to nitpick about, but a very concrete concern, and that some of the DFT-MD results presented in literature might suffer from this problem.

c) Please see our response b).
